# Supplementary material for: A H2O2-activatable nanoprobe for diagnosing interstitial cystitis and liver ischemia-reperfusion injury via multispectral optoacoustic tomography and NIR-II fluorescent imaging
Source: Nat Commun. 2021 Nov 25;12:6870. doi: 10.1038/s41467-021-27233-4 (PMC8617030; doi:10.1038/s41467-021-27233-4)
Supplement: Supplementary file 1 — Supplementary Information [file 41467_2021_27233_MOESM1_ESM.pdf]

Supplementary Information

A H_2O_2-Activatable Nanoprobe for Diagnosing Interstitial Cystitis and Liver Ischemia-Reperfusion Injury via Multispectral Optoacoustic Tomography and NIR-II Fluorescent Imaging

Chen et al.

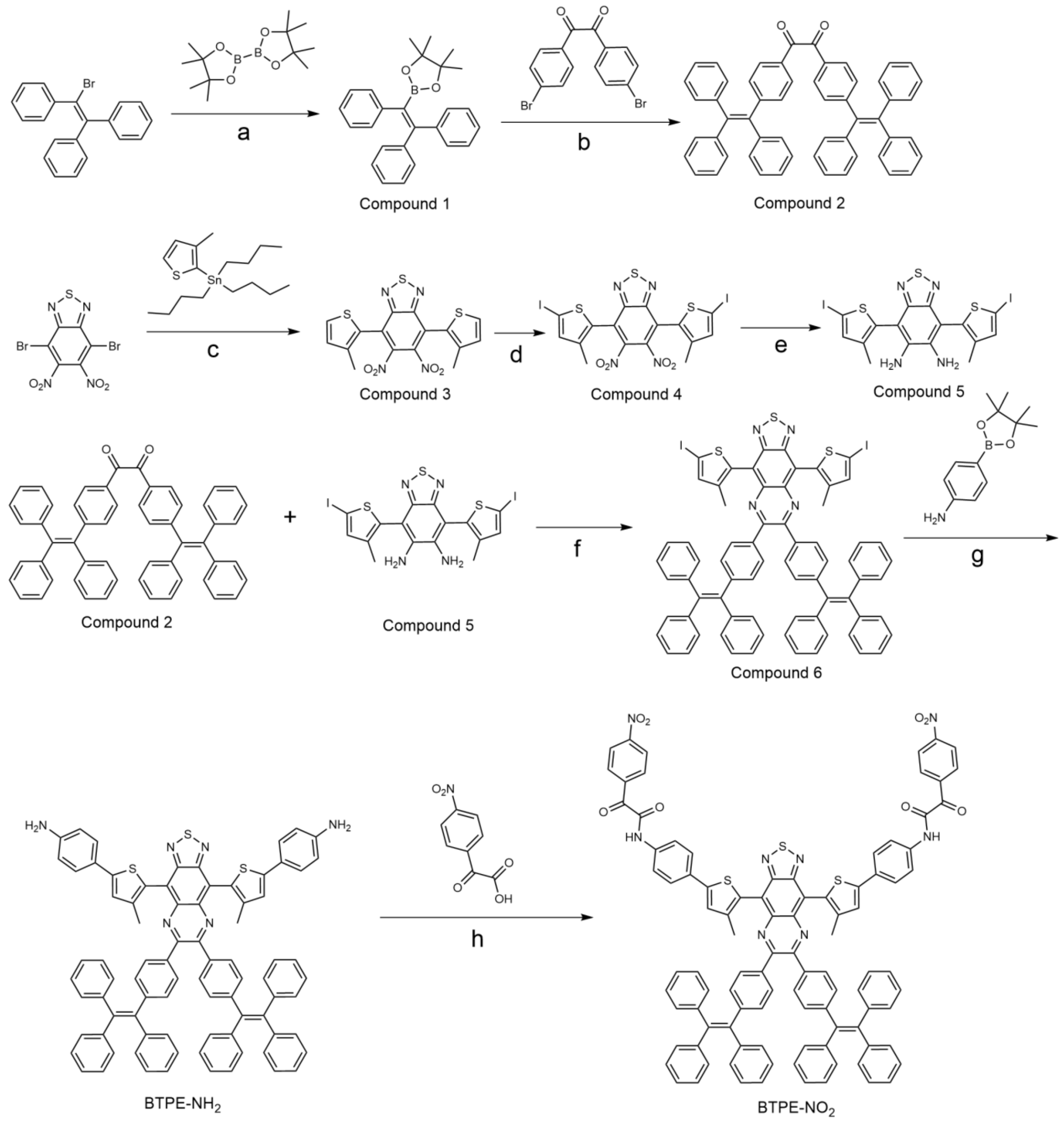

Supplementary Fig. 1. Synthesis route of the probe compound BTPE-NO_2 and the chromophore BTPE-NH_2. Reagents, conditions and yields: **a)** $Pd(PPh_3)_2Cl_2$, K_2CO_3 (2 M, aqueous solution), 1,4-dioxane, N_2, 90 °C, 48 h, 96%; **b)** $Pd(PPh_3)_4$, K_2CO_3 (2 M, aqueous solution), Tetrahydrofuran, N_2, 80 °C, 24 h, 87%; **c)** $Pd(PPh_3)_4$, Tetrahydrofuran, N_2, 80 °C, 24 h, 96%; **d)** NIS, Chloroform, Acetic acid, 40 °C, 24 h, 98%; **e)** Fe, Acetic acid, 80 °C, overnight, 96%; **f)** Acetic acid, 110 °C, 24 h, N_2, 97%; **g)** $Pd(PPh_3)_4$, K_2CO_3 (2 M, aqueous solution), Tetrahydrofuran, N_2, 24 h, 69%; **h)** 1) Oxalyl Chloride, Dichloromethane, N,N-Dimethylformamide, N_2, 45 °C, 1 h; 2) Triethylamine, Dichloromethane, Room temperature, 1 h, 44%.

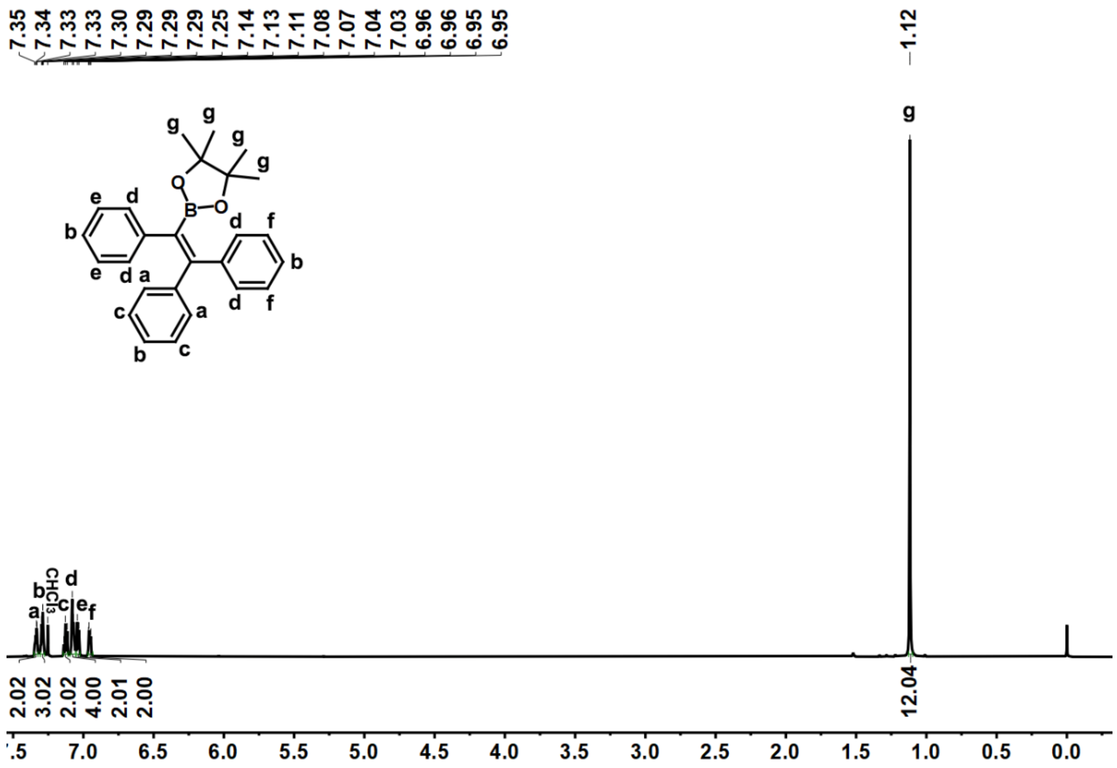

Supplementary Fig. 2. ^{1}H NMR spectrum of compound 1 in $CDCl_3$.

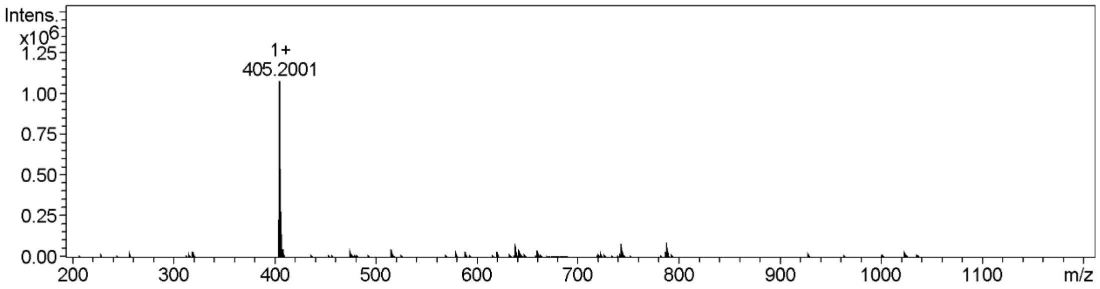

Supplementary Fig. 3. HR mass spectrum of compound 1.

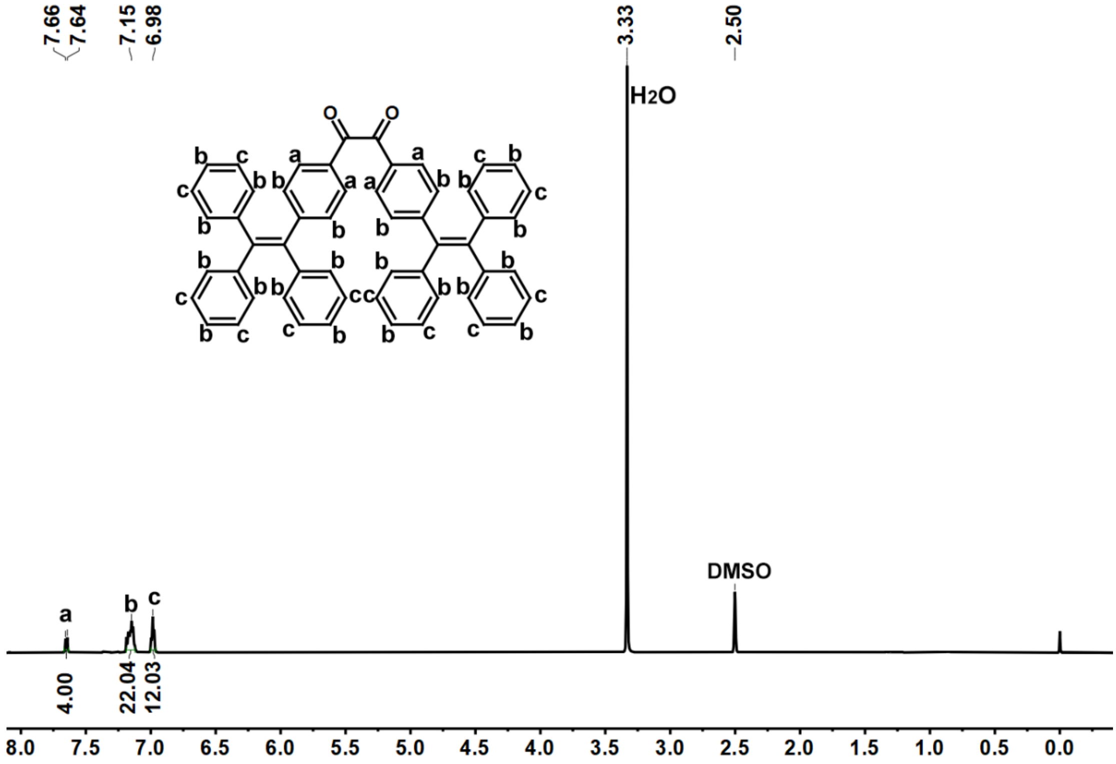

Supplementary Fig. 4. ^{1}H NMR spectrum of compound 2 in DMSO-d_6.

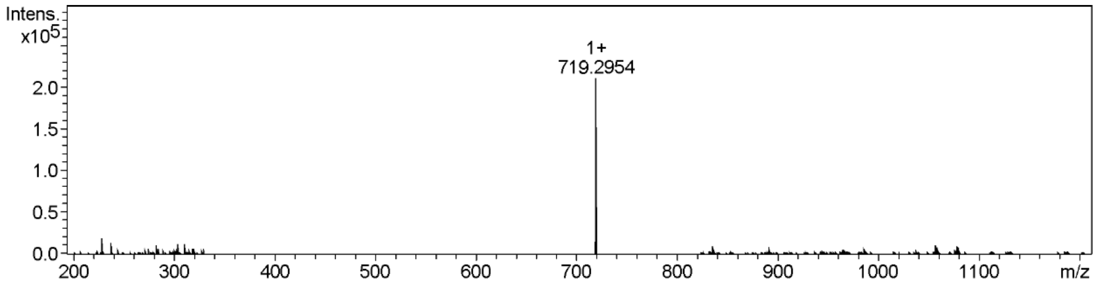

Supplementary Fig. 5. HR mass spectrum of compound 2.

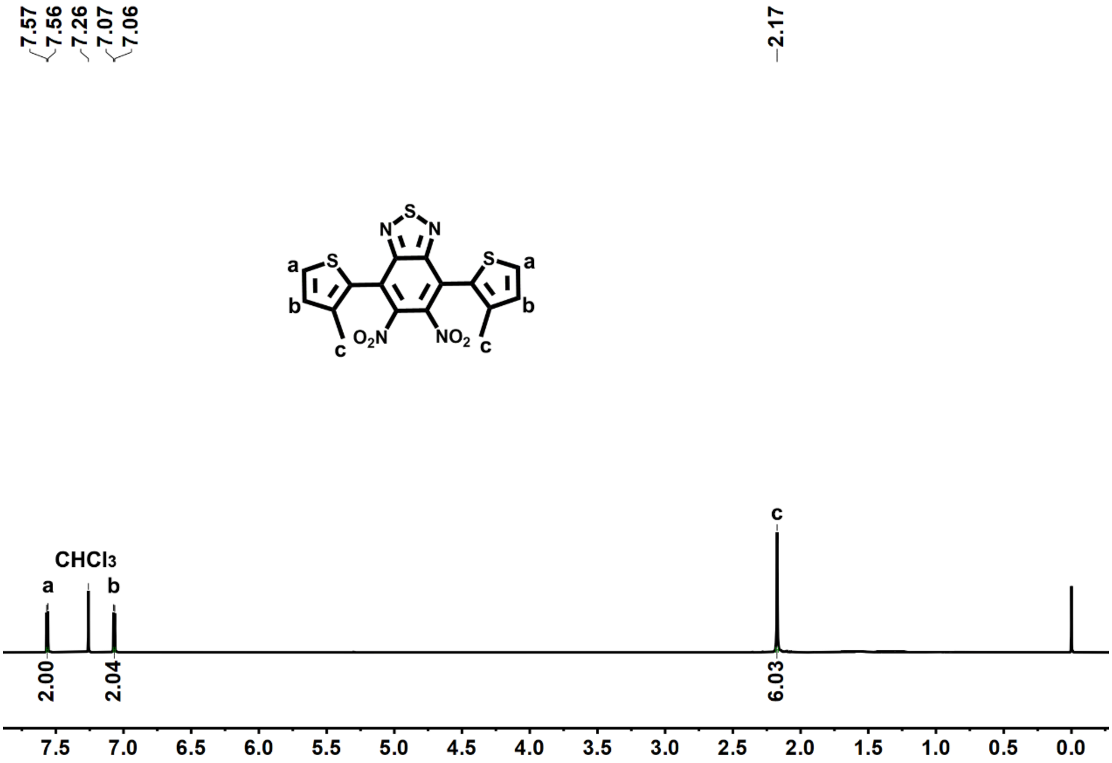

Supplementary Fig. 6. ^{1}H NMR spectrum of compound 3 in CDCl3.

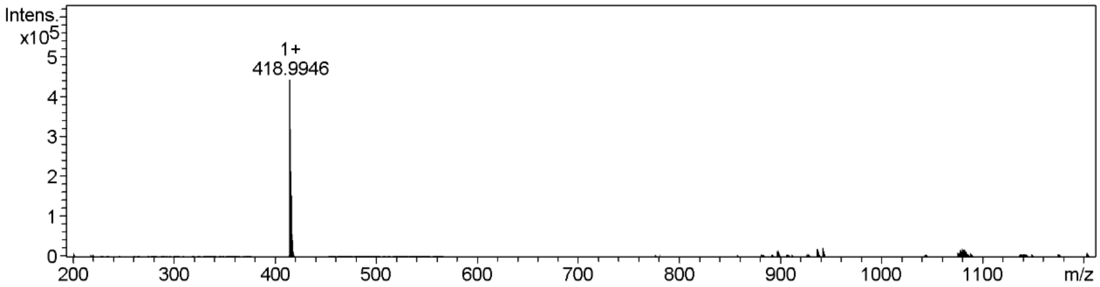

Supplementary Fig. 7. HR mass spectrum of compound 3.

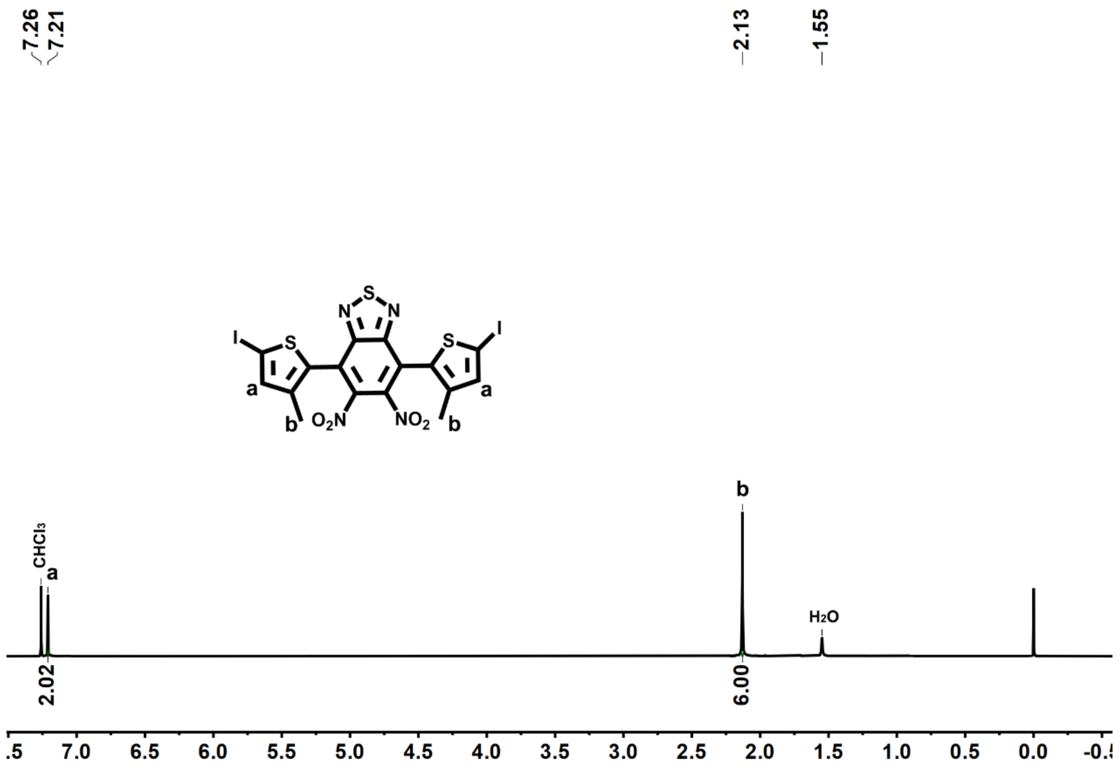

Supplementary Fig. 8. ^{1}H NMR spectrum of compound 4 in $CDCl_3$.

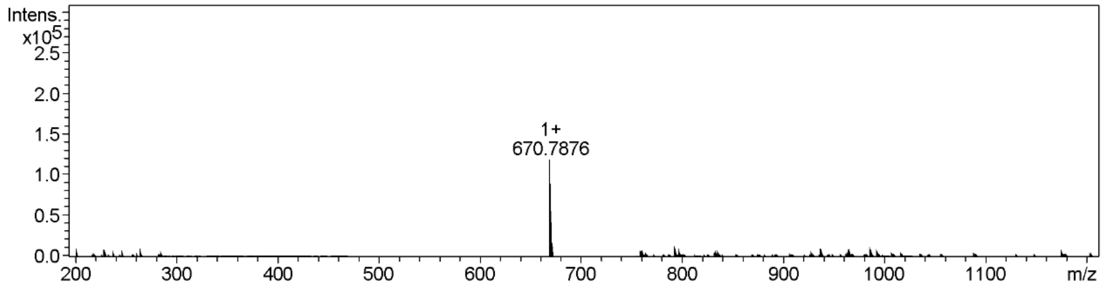

Supplementary Fig. 9. HR mass spectrum of compound 4.

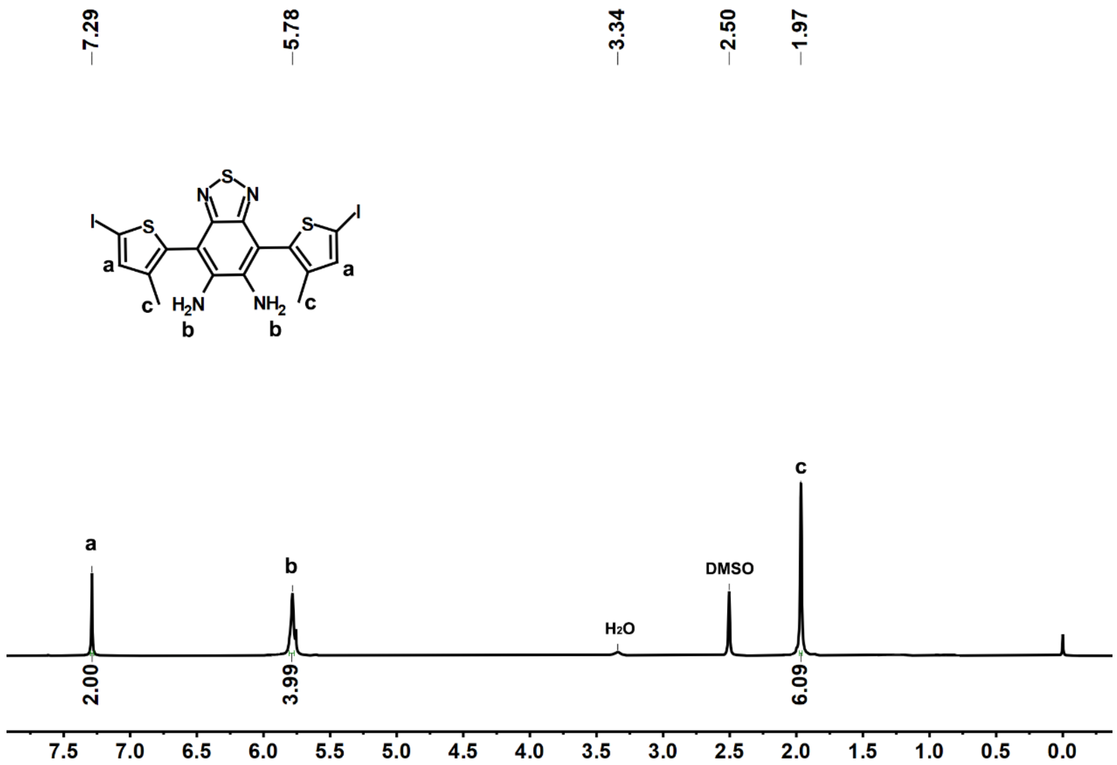

Supplementary Fig. 10. ^{1}H NMR spectrum of compound 5 in DMSO-d_6.

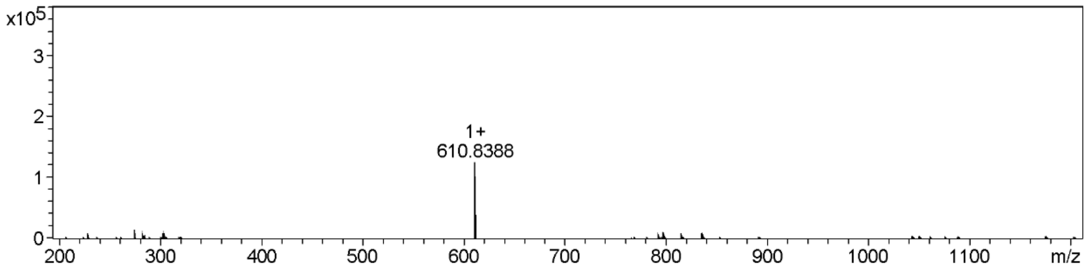

Supplementary Fig. 11. HR mass spectrum of compound 5.

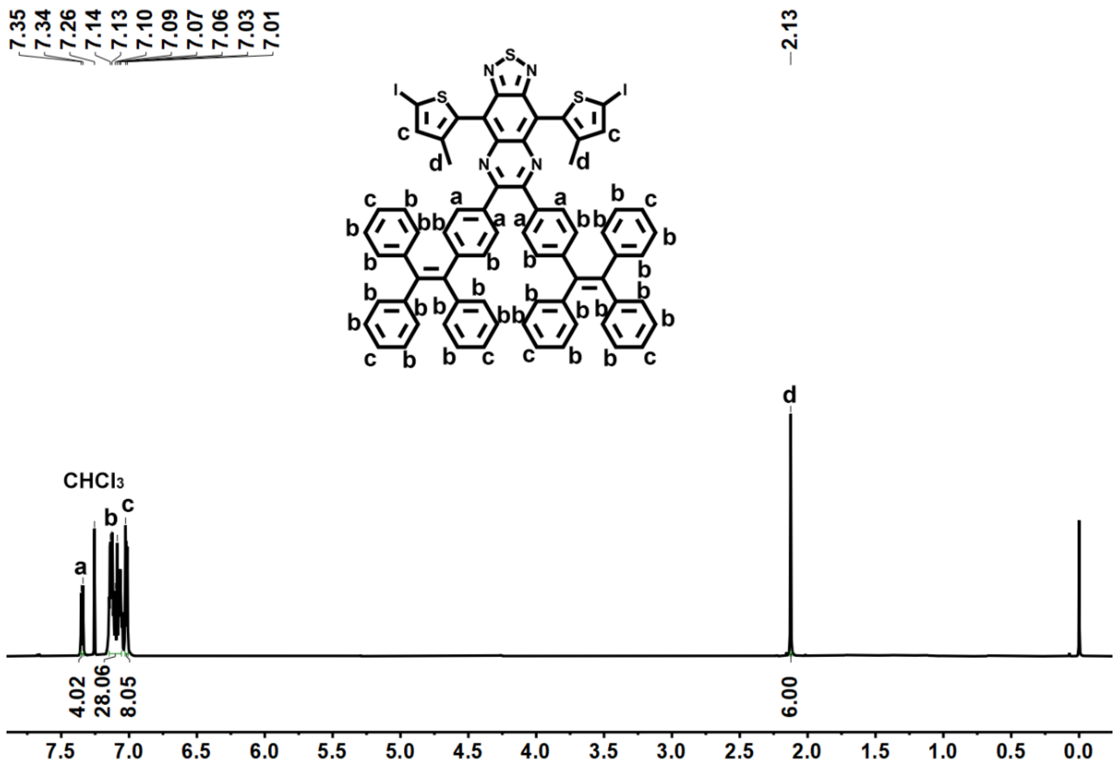

Supplementary Fig. 12. ^{1}**H NMR spectrum of compound 6 in** $CDCl_3$**.**

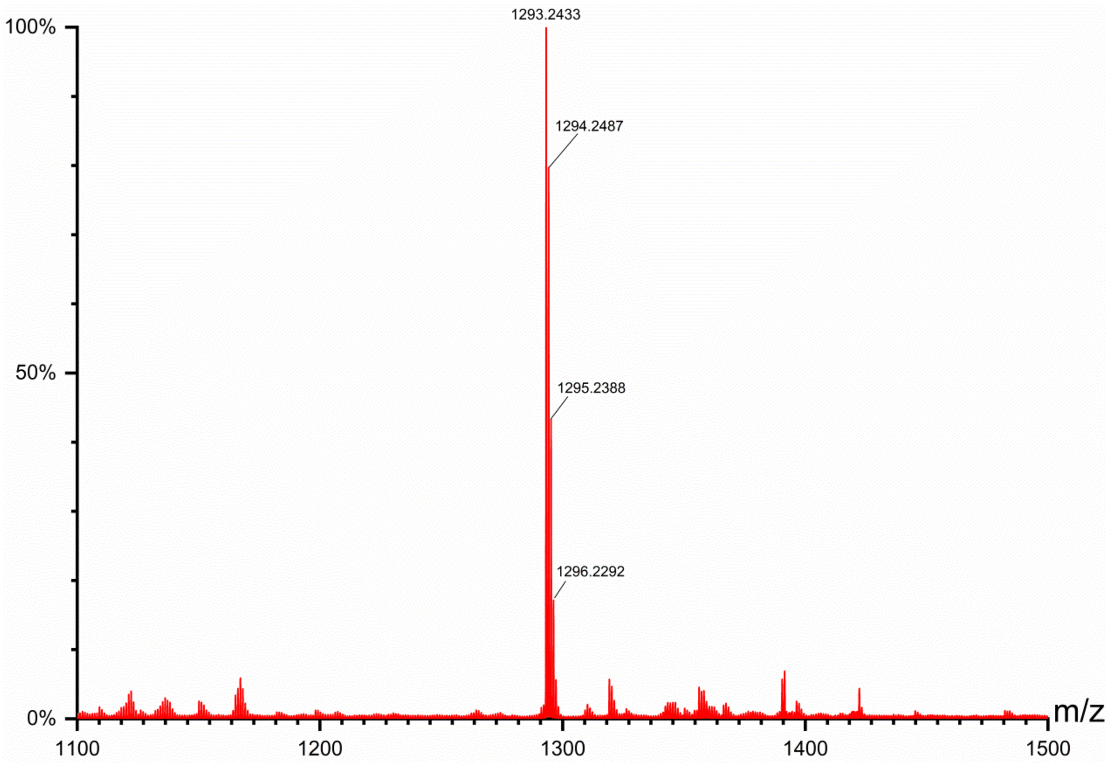

Supplementary Fig. 13. MALDI TOF mass spectrum of compound 6.

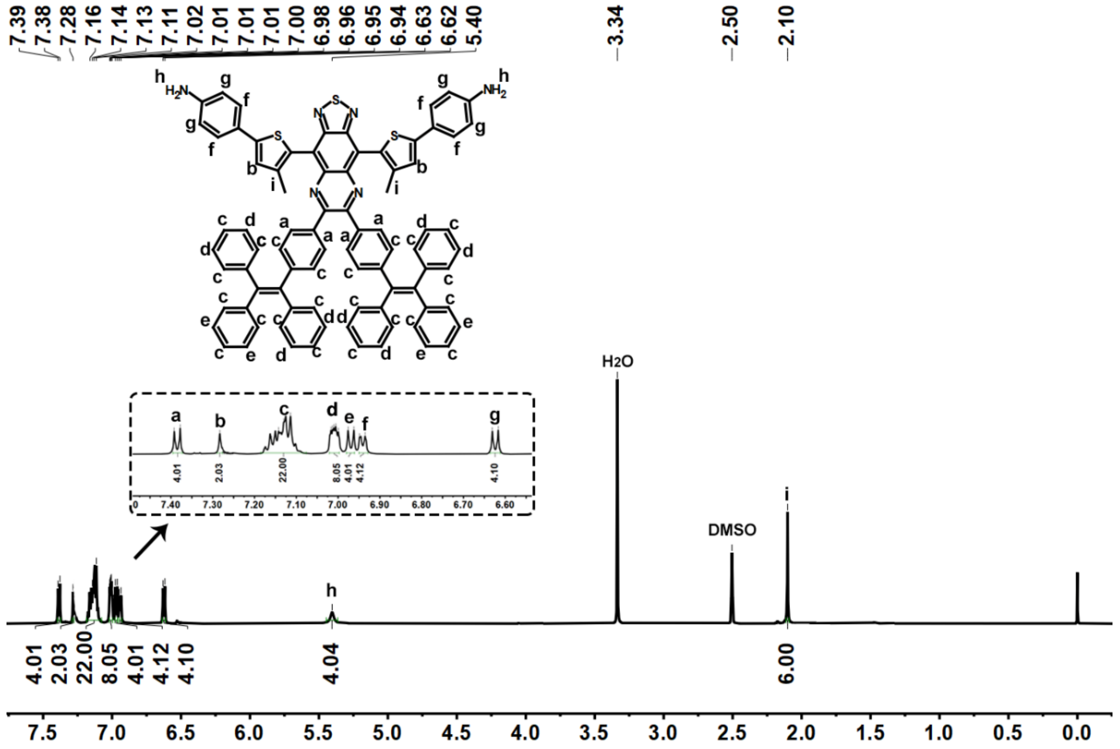

Supplementary Fig. 14. ^{1}H NMR spectrum of BTPE-NH_2 in DMSO-d_6.

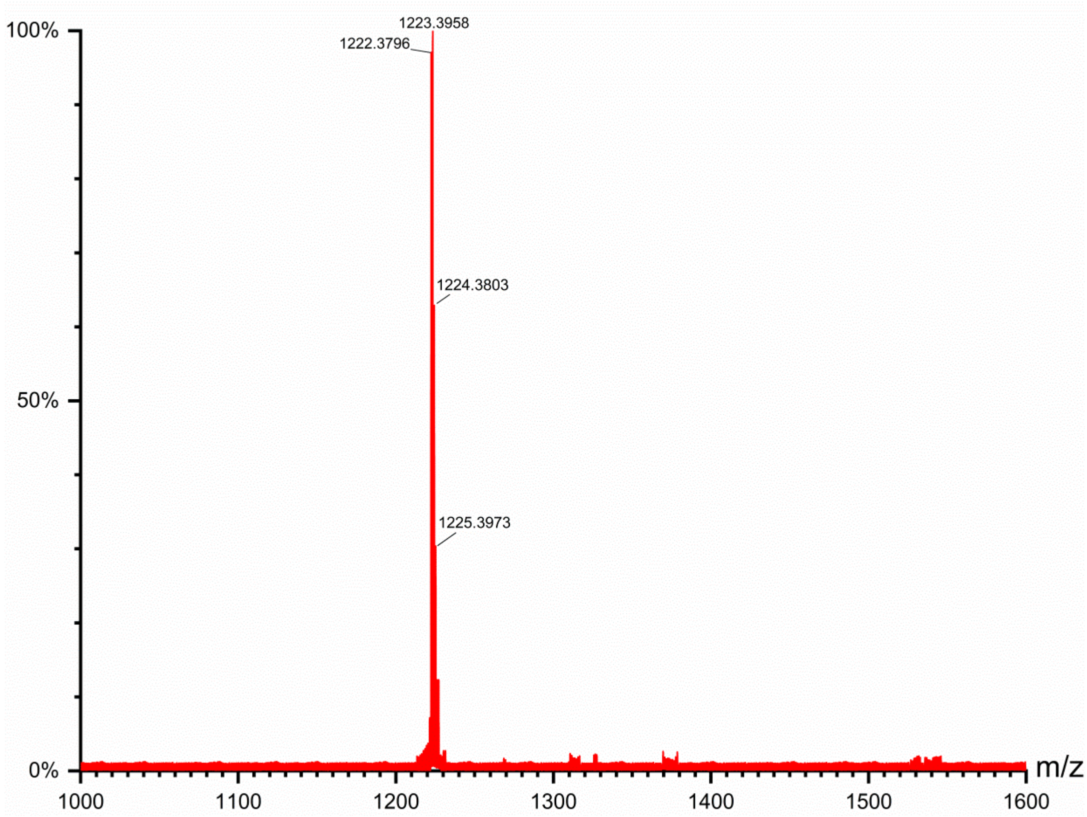

Supplementary Fig. 15. MALDI TOF mass spectrum of BTPE-NH_2.

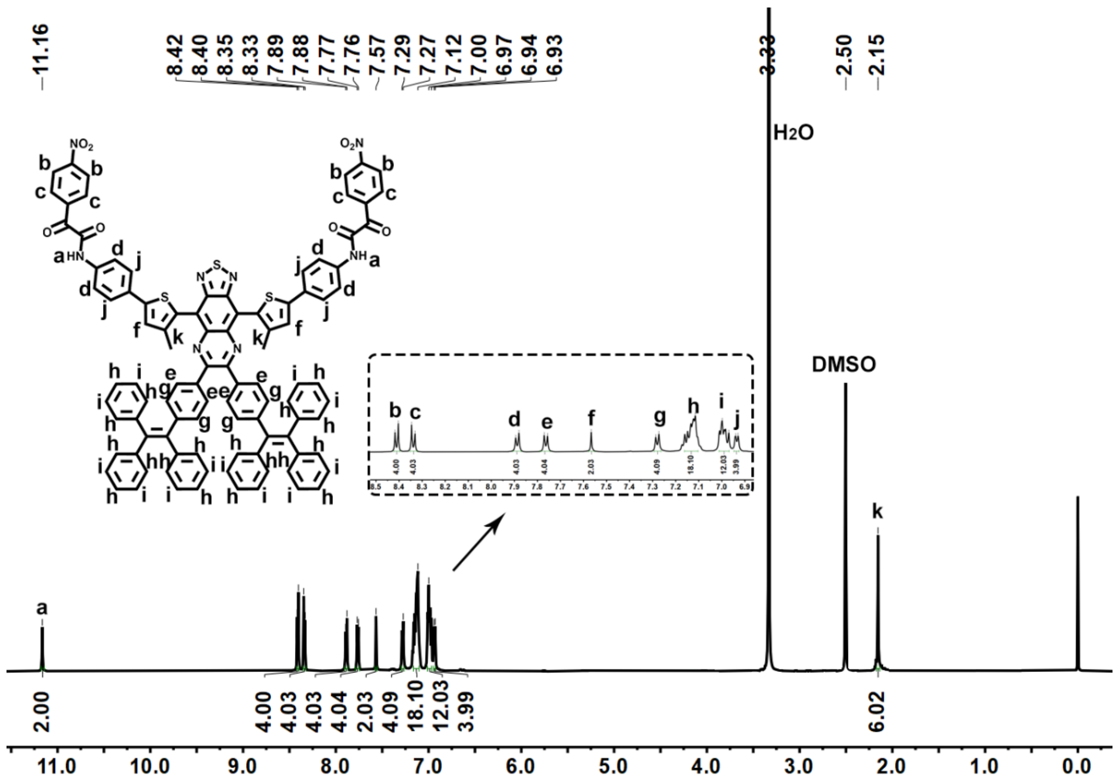

Supplementary Fig. 16. ^{1}H NMR spectrum of BTPE-NO$_2$ in DMSO-d_6.

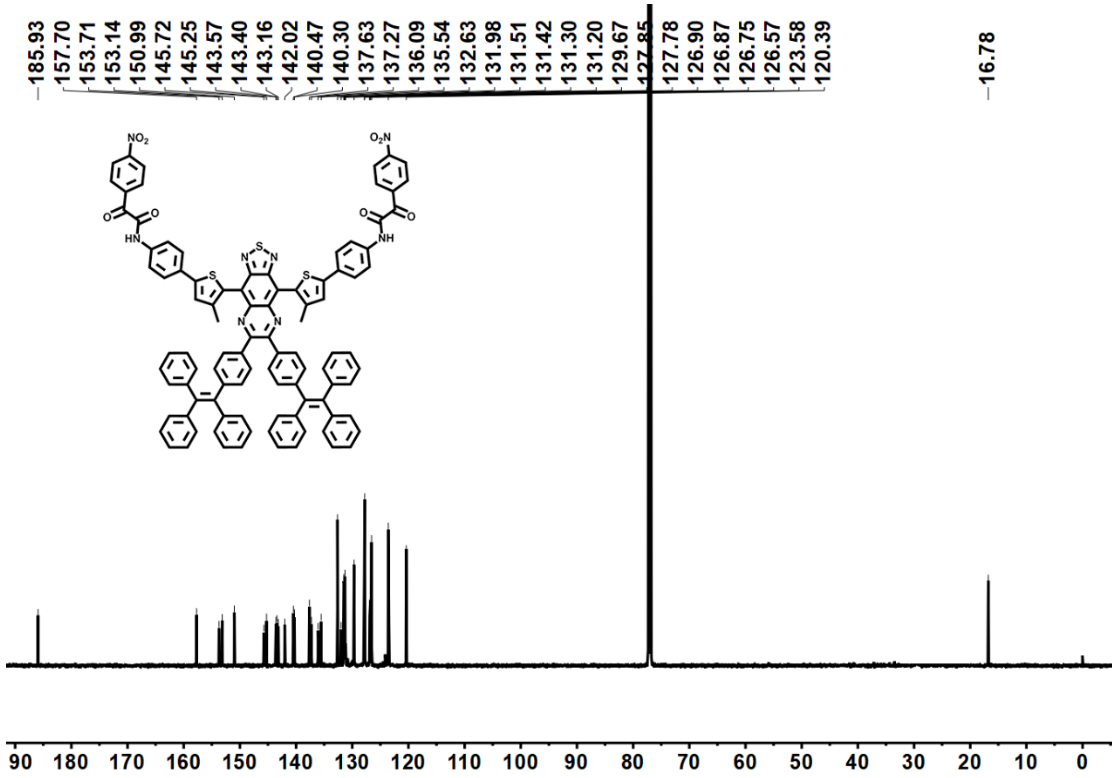

Supplementary Fig. 17. ^{13}C NMR spectrum of BTPE-NO$_2$ in DMSO-d_6.

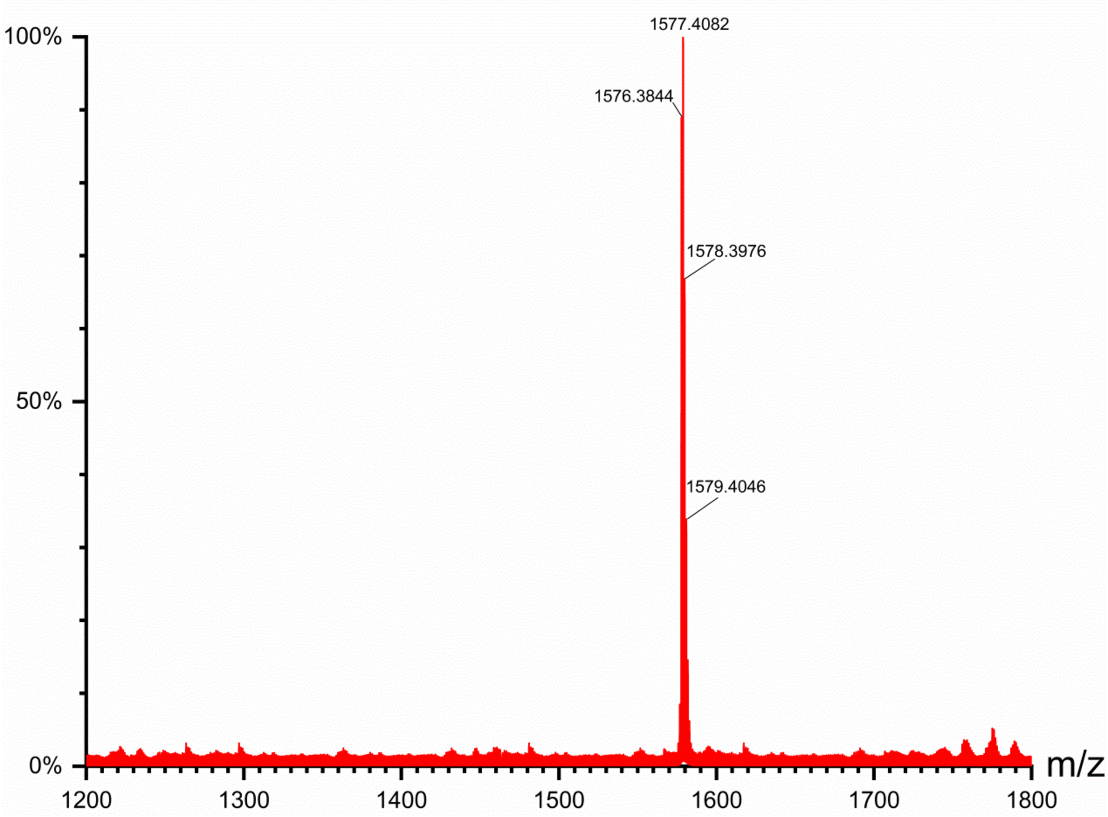

Supplementary Fig. 18. MALDI TOF mass spectrum of BTPE-NO$_2$.

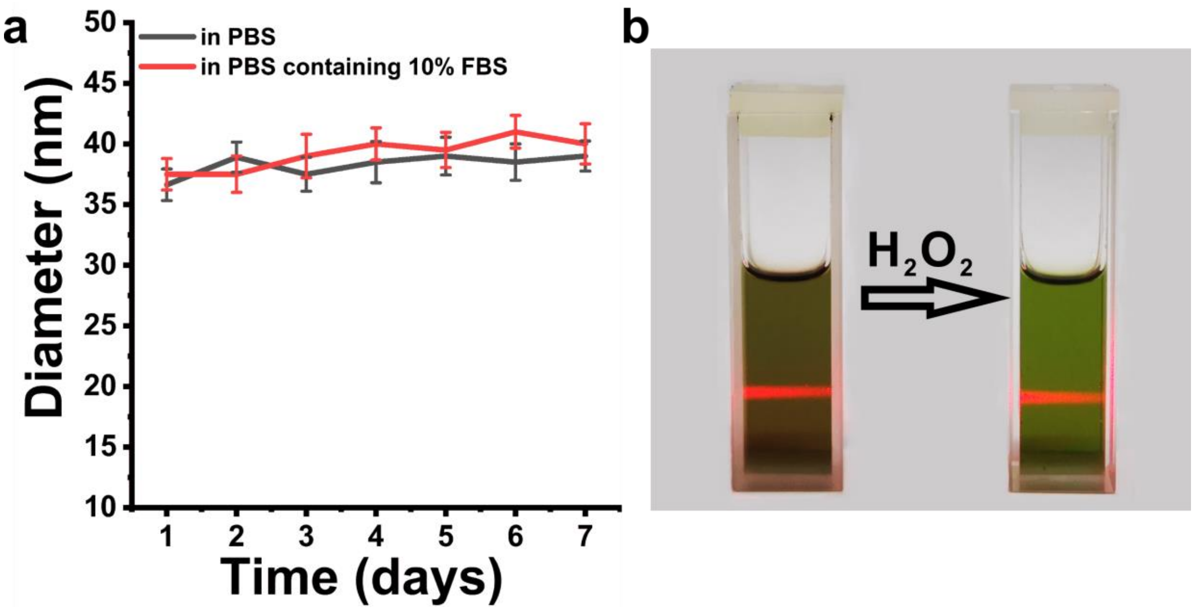

Supplementary Fig. 19. Particle size stability test and Tyndall phenomenon of nanoparticles. (a) Average diameter of the nanoprobe BTPE-NO$_2$@F127 dispersion in PBS (pH = 7.4, 10 mM) or in PBS containing 10% FBS at room temperature for 7 days. n = 3 independent samples. Data are presented as mean values +/- SD. (b) Photographs of BTPE-NO$_2$@F127 nanoparticle dispersion before and after incubation with H_2O_2 showing Tyndall phenomenon.

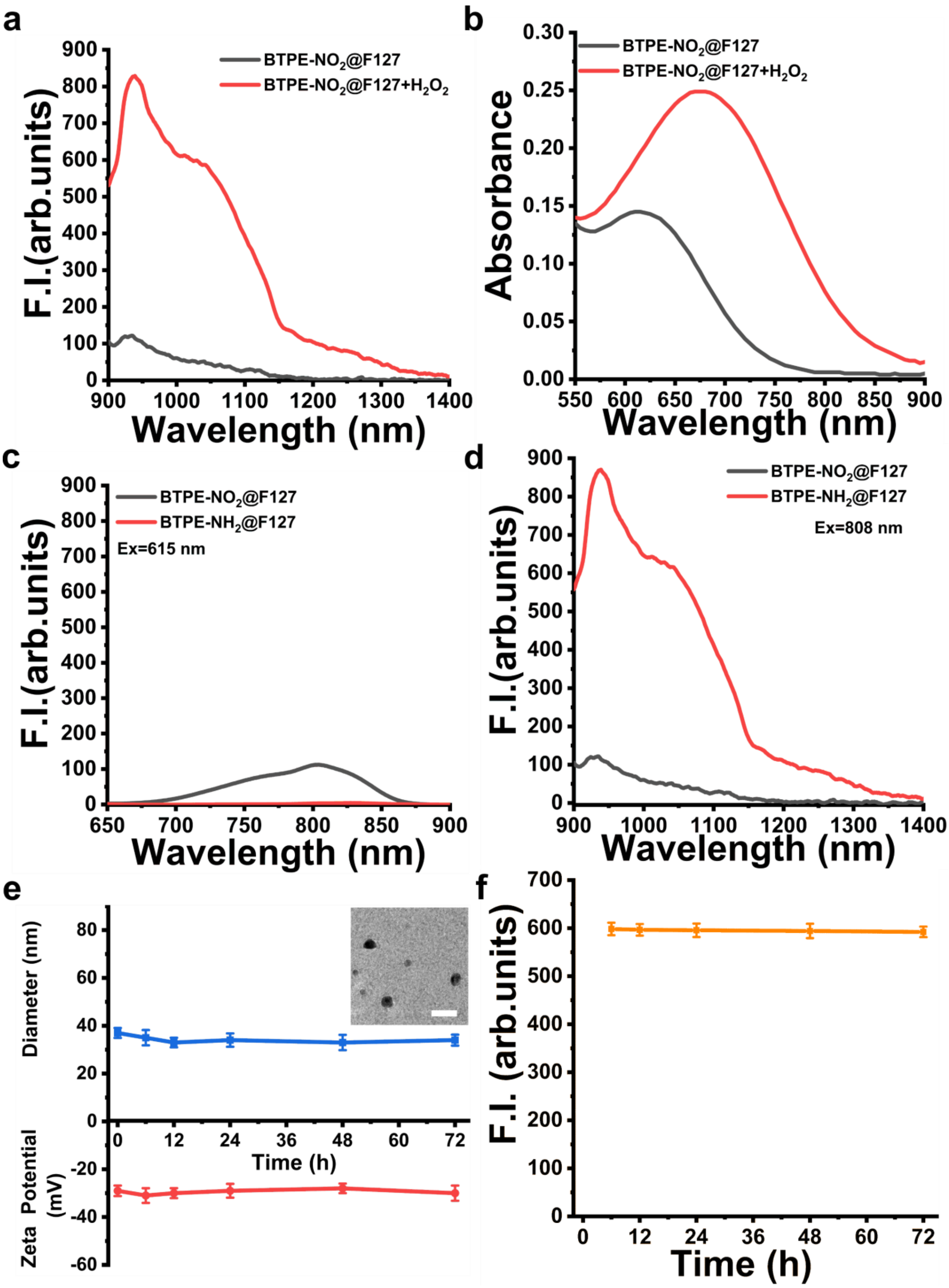

Supplementary Fig. 20. Optical test and stability test after responding to H_2O_2. (a) NIR-II fluorescence spectra for the nanoprobe BTPE-NO_2@F127 (BTPE-NO_2 32.6 μg mL^{-1}) with or without incubation of 100 μM H_2O_2. (b) Absorption spectra for the nanoprobe BTPE-NO_2@F127 (BTPE-NO_2 32.6 μg mL^{-1}) with or without incubation of 100 μM H_2O_2. (c)

NIR-I fluorescence spectra for the nanoprobe BTPE-NO_2@F127 (BTPE-NO_2 32.6 μg mL^{-1}) or the BTPE-NH_2@F127(BTPE-NH_2 25.3 μg mL^{-1}) with the excitation of 615 nm. (d) NIR-II fluorescence spectra for the nanoprobe BTPE-NO_2@F127(BTPE-NO_2 32.6 μg mL^{-1}) or the BTPE-NH_2@F127(BTPE-NH_2 25.3 μg mL^{-1}) with the excitation of 808 nm. (e) Average diameter and zeta potential of the BTPE-NO_2@F127 after responding to H_2O_2 as a function of time for 72 h. Inset: representation of the TEM images for the BTPE-NO_2@F127 after responding to H_2O_2 at 72 h. Scale bar: 50 nm. n = 3 independent samples. Data are presented as mean values +/- SD. (f) Change of fluorescent intensity at 1028 nm of the nanoprobe BTPE-NO_2@F127 after responding to H_2O_2 for 72 h. n = 3 independent samples. Data are presented as mean values +/- SD. F.I.: fluorescence intensity. The NIR-I fluorescence spectra were recorded on Hitachi F-4700 fluorescence spectrophotometer (excitation: 615 nm). The near-infrared II (NIR-II) fluorescence spectra were recorded on NIRQUEST512 spectrometer (excitation: 808 nm laser, emission range: 900-1700 nm).

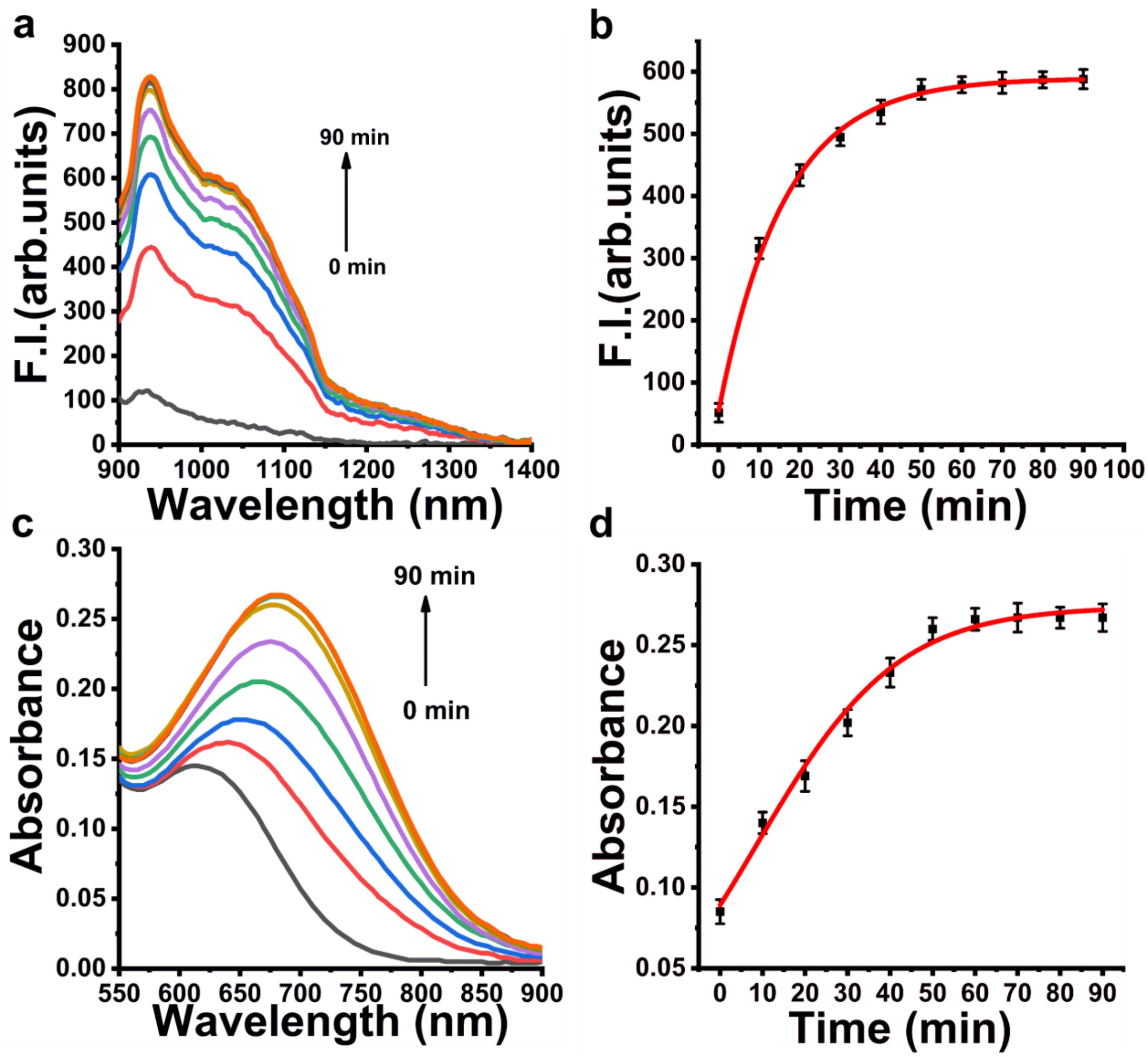

Supplementary Fig. 21. Time-dependent optical response test. (a) Time-dependent NIR-II fluorescence spectra of the nanoprobe BTPE-NO_2@F127 (BTPE-NO_2 32.6 μg mL^{-1}) in PBS (pH 7.4) at different time points under incubation with 100 μM H_2O_2 at 37 °C. (b) Fluorescent intensity at 1028 nm at different time points after incubation with 100 μM H_2O_2 at 37 °C. n = 3 independent samples. Data are presented as mean values +/- SD. (c) Time-dependent absorption spectra of the nanoprobe BTPE-NO_2@F127 (BTPE-NO_2 32.6 μg mL^{-1}) in PBS (pH 7.4) at different time points under incubation with 100 μM H_2O_2 at 37 °C. (d) Absorbance at 680 nm at different time points after incubation with 100 μM H_2O_2. n = 3 independent samples. Data are presented as mean values +/- SD. F.I.: fluorescence intensity.

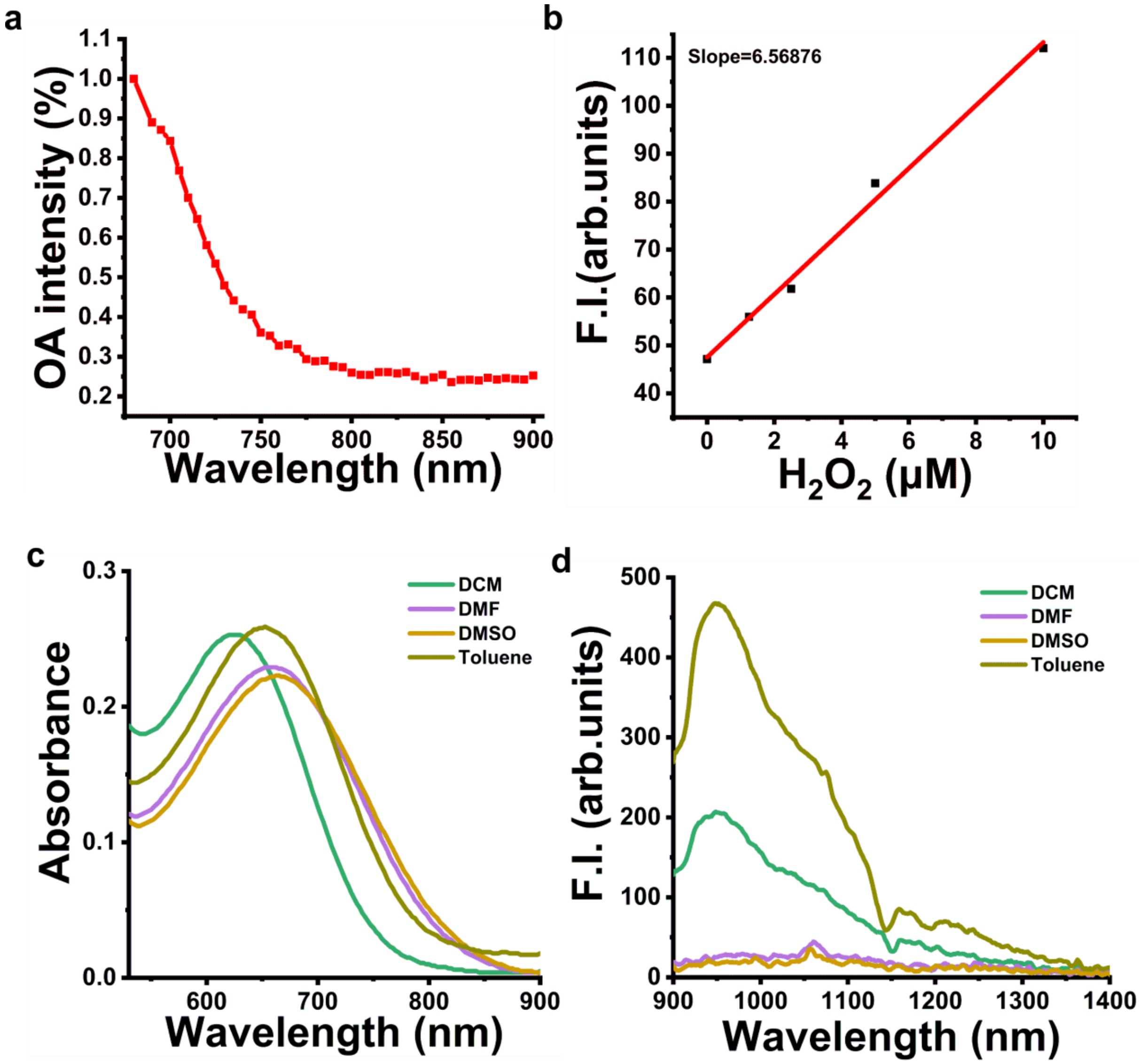

Supplementary Fig. 22. Normalized optoacoustic intensities, detection limit and spectral test in different organic solvents. (a) Normalized optoacoustic intensities for the nanoprobe BTPE-NO_2@F127 (BTPE-NO_2 32.6 μg mL^{-1}) upon incubation with 100 μM H_2O_2 in PBS (pH = 7.4) at 37 °C for 90 min as a function of excitation wavelength. (b) Fluorescence intensity at 1028 nm as a function of H_2O_2 in the low concentration range. (c) Absorption spectra for the BTPE-NH_2 in the different organic solvents (DCM: dichloromethane, DMF: dimethylformamide, DMSO: dimethyl sulfoxide). (d) NIR-II fluorescence spectra for the BTPE-NH_2 in the different organic solvents. F.I.: fluorescence intensity; OA: optoacoustic.

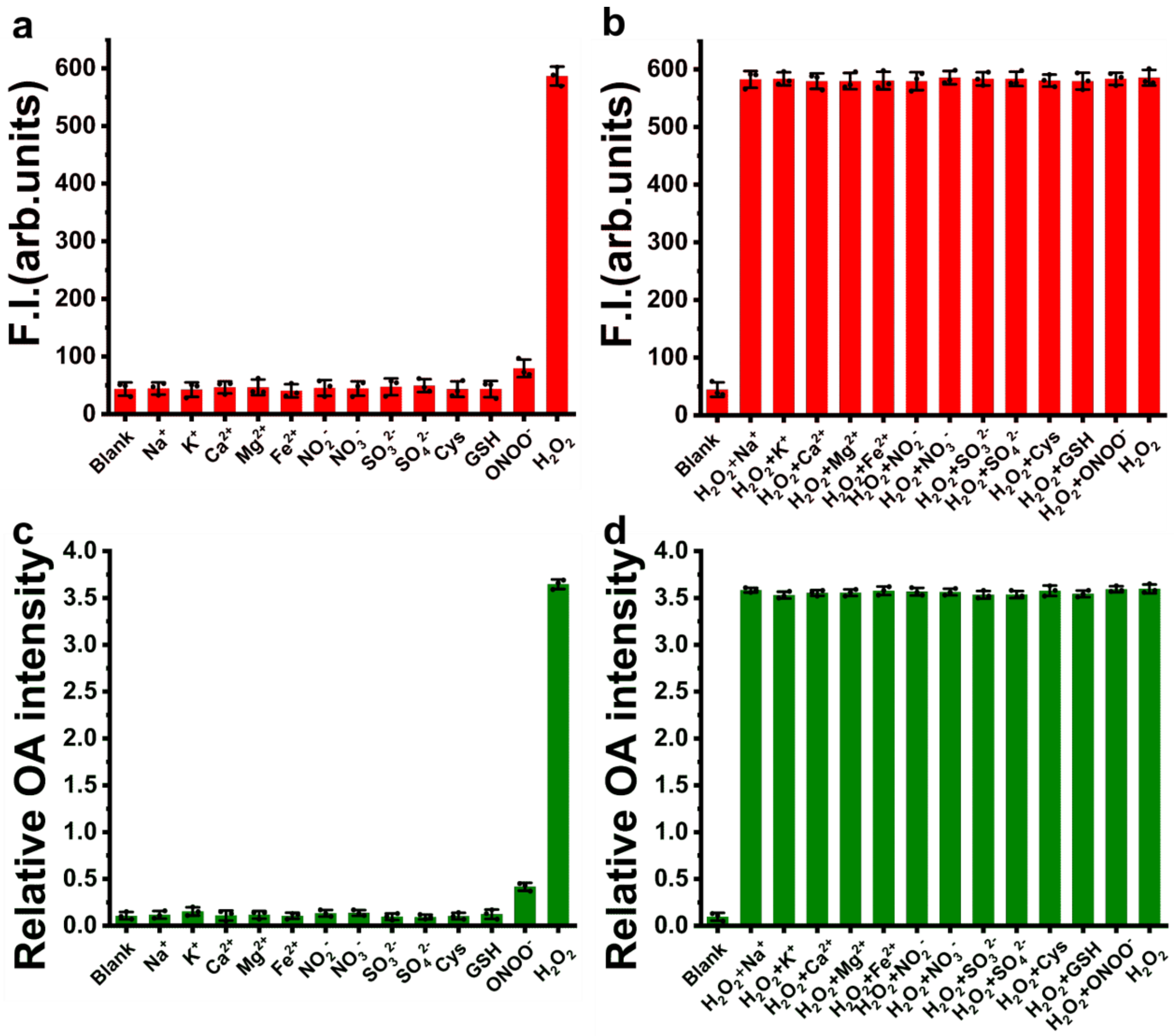

Supplementary Fig. 23. Selectivity and anti-interference test. (a) Fluorescent intensity of the nanoprobe BTPE-NO_2@F127 (BTPE-NO_2 30 µg mL^{-1}) at 1028 nm upon incubation with different substances in PBS (pH = 7.4) at 37 °C for 90 min (cations and anions: 1 mM; GSH: 10 mM; Cys: 200 µM; $ONOO^-$: 100 µM). (b) Fluorescent intensity of the nanoprobe BTPE-NO_2@F127 (BTPE-NO_2 30 µg mL^{-1}) at 1028 nm upon treatment with 100 µM H_2O_2 and simultaneously in the presence of individual potential interferent respectively for 90 min in PBS (pH = 7.4) at 37 °C (cations and anions: 1 mM; GSH: 10 mM; Cys: 200 µM; $ONOO^-$: 100 µM). (c) Relative optoacoustic intensity of the nanoprobe BTPE-NO_2@F127 (BTPE-NO_2 32.6 µg mL^{-1}) in the presence of different substances respectively for 90 min in PBS (pH = 7.4) at 37 °C (cations and anions: 1 mM; GSH: 10 mM; Cys: 200 µM; $ONOO^-$: 100 µM). (d) Relative optoacoustic intensity of the nanoprobe BTPE-NO_2@F127 (BTPE-NO_2 32.6 µg mL^{-1}) in the presence of 100 µM H_2O_2 and simultaneously in the presence of individual potential interferent respectively for 90 min in PBS (pH = 7.4) at 37 °C (cations and anions: 1 mM; GSH: 10 mM; Cys: 200 µM; $ONOO^-$: 100 µM). n = 3 independent samples. Data are presented as mean values +/- SD. F.I.: fluorescence intensity; OA: optoacoustic.

a

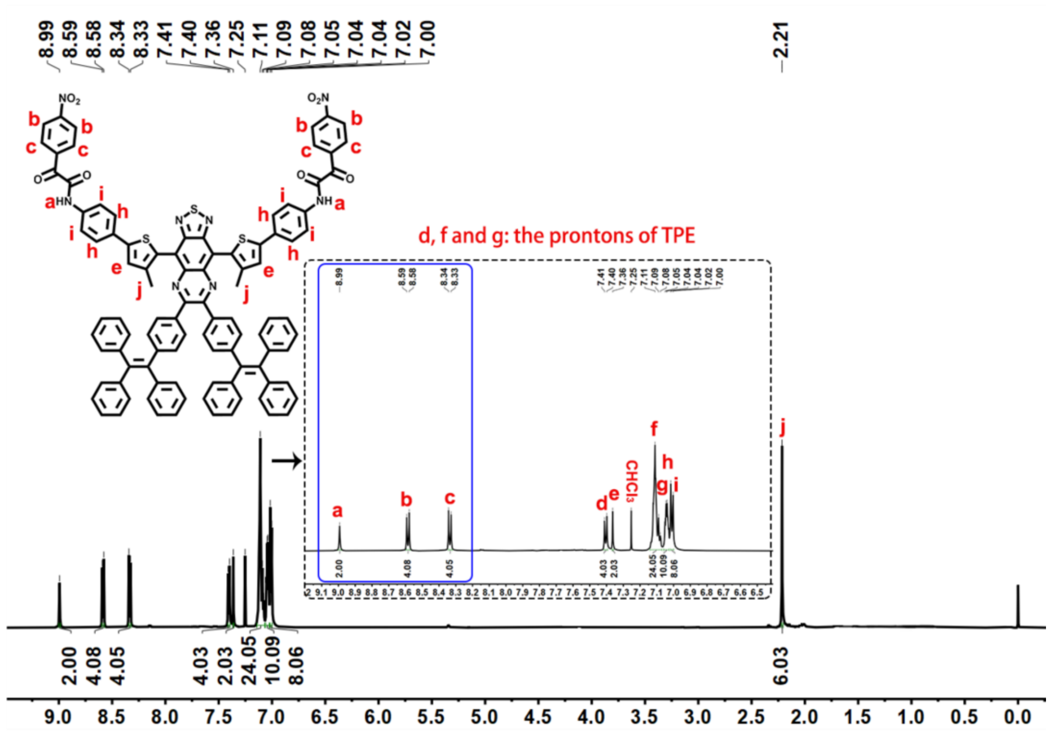

b

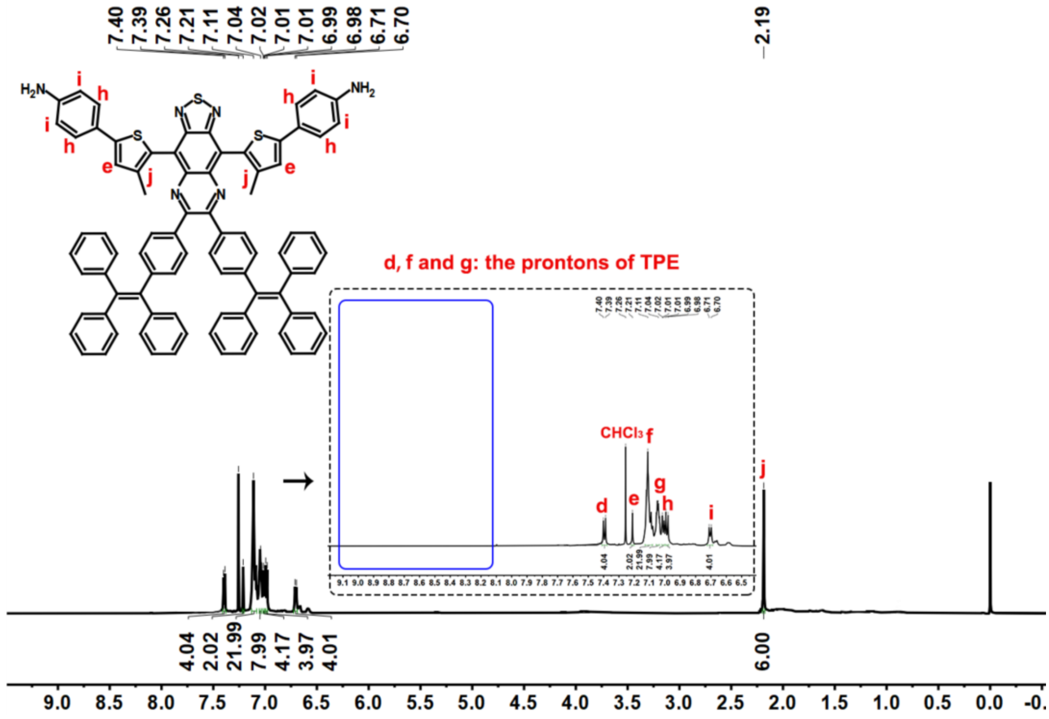

Supplementary Fig. 24. NMR characterization before and after the response. (a) ^{1}H NMR spectrum of the probe compound (BTPE-NO_2) before responding to H_2O_2. (b) ^{1}H NMR spectrum of the probe compound after being incubated with H_2O_2 for 90 min in PBS (pH = 7.4) at 37 °C (complete reaction, the reaction product was purified by silica-gel chromatography column). TPE: tetraphenylethylene.

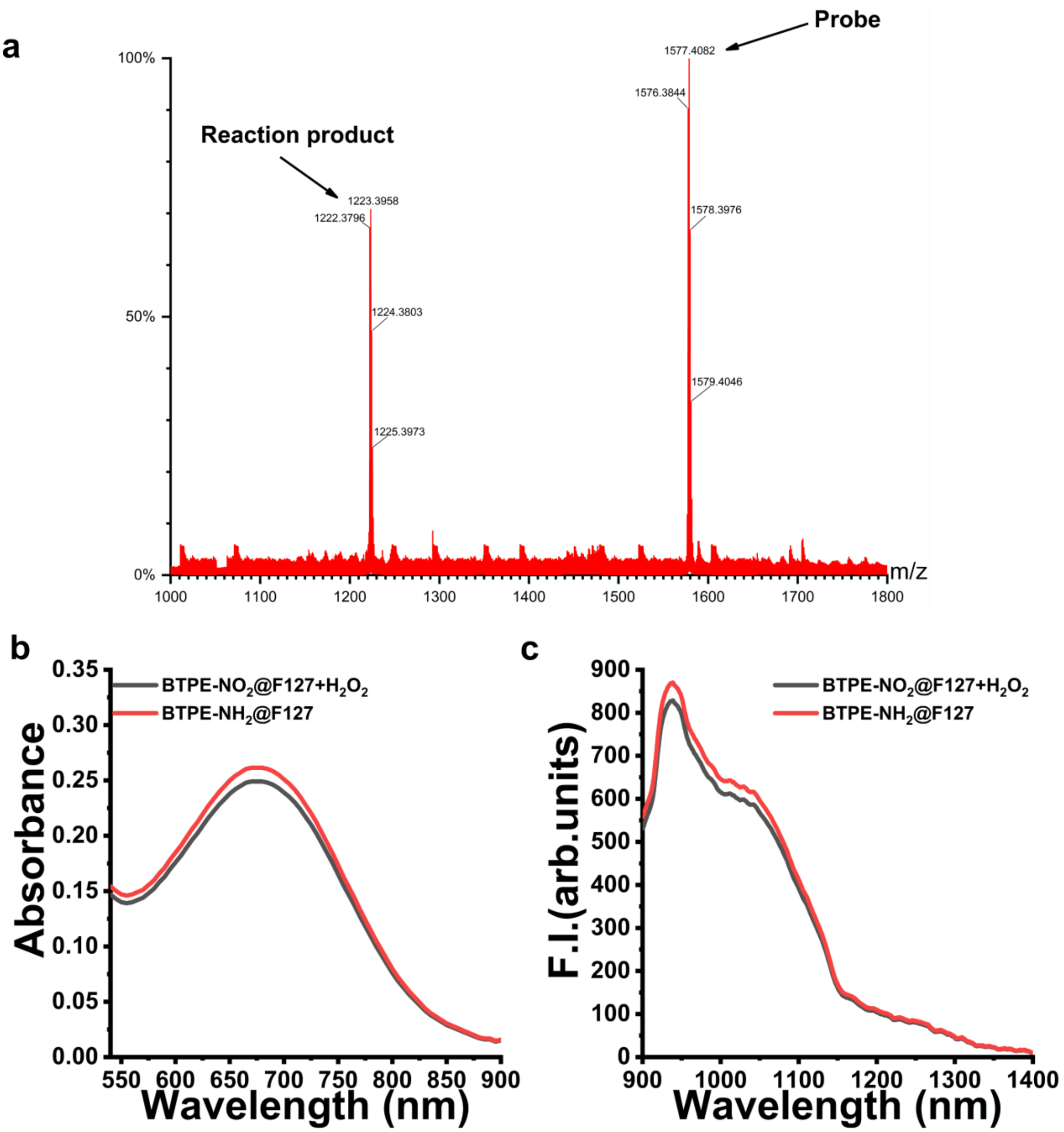

Supplementary Fig. 25. Mass spectrum and spectral characterization before and after the response. (a) MALDI TOF mass spectrum of the probe compound after being incubated with H_2O_2 for 30 min at 37 °C in PBS (pH = 7.4) (incomplete reaction, the reaction product was precipitated in diethyl ether and dissolved in tetrahydrofuran for measurement). (b) Absorption spectra for the probe BTPE-NO_2@F127 (BTPE-NO_2 32.6 μg mL^{-1}) with incubation of 100 μM H_2O_2 and for the BTPE-NH_2@F127 (BTPE-NH_2 25.3 μg mL^{-1}). (c) NIR-II fluorescence spectra for the probe BTPE-NO_2@F127 (BTPE-NO_2 32.6 μg mL^{-1}) with incubation of 100 μM H_2O_2 and for the BTPE-NH_2@F127 (BTPE-NH_2 25.3 μg mL^{-1}). F.I.: fluorescence intensity.

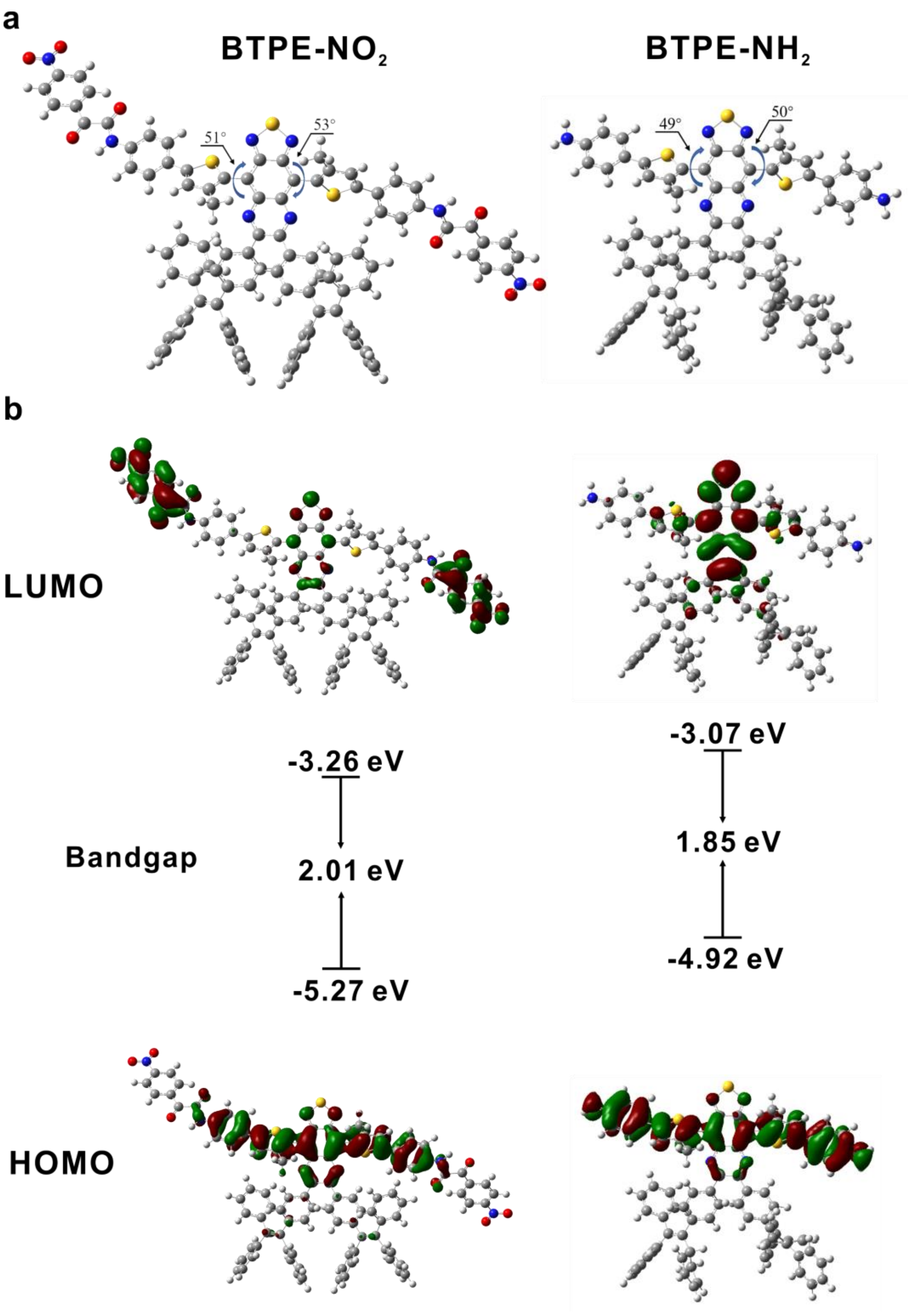

Supplementary Fig. 26. Theoretical calculations. (a) Optimized S_0 (ground state) geometries of BTPE-NO_2 and BTPE-NH_2 in dichloromethane with IEFPCM solvent model. (b) HOMO and LUMO orbital surfaces of BTPE-NO_2 and BTPE-NH_2 in the geometrically optimized structure using B3LYP/6-31G (d,p) scrf = (cpcm, solvent = Dichloromethane) in Gaussian 16 W program. LUMO: lowest unoccupied molecular orbital; HOMO: highest occupied molecular orbital.

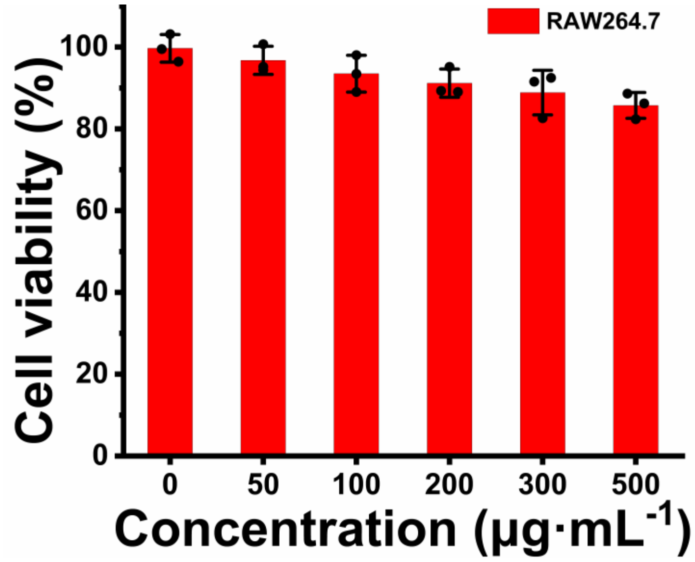

Supplementary Fig. 27. Cell viability test. Cell viability for RAW264.7 cells in the presence of the nanoprobe BTPE-NO_2@F127 at varied concentrations. Three independent experiments were conducted, and for each independent experiment, the assays were conducted in eight replicates. Data are presented as mean values +/- SD.

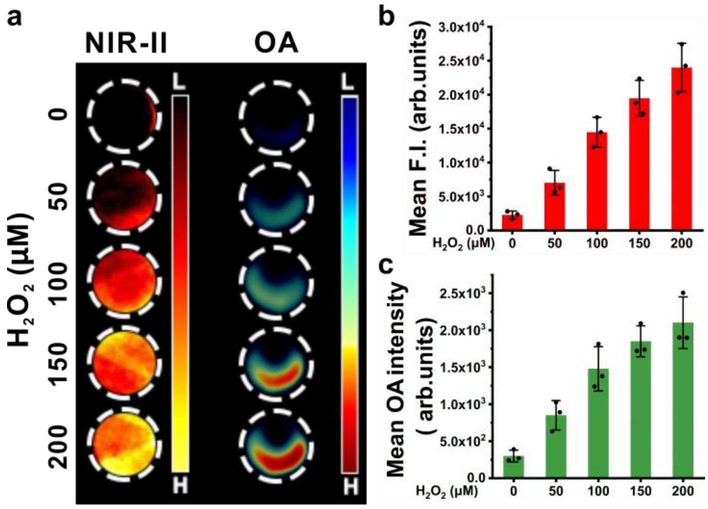

Supplementary Fig. 28. Cell imaging experiments. (a) NIR-II fluorescent images and optoacoustic images for RAW264.7 cells incubated with varied concentrations of H_2O_2 at 37 °C in the presence of the nanoprobe BTPE-NO_2@F127 (BTPE-NO_2 37.2 μg mL^{-1}). For NIR-II fluorescence imaging: excitation wavelength: 808 nm, emission filter: 900-1700 nm, color bar: L: 6.0×10^2, H: 6.0×10^4 (arb. units). For optoacoustic imaging: excitation at 680 nm, color bar: L: 6.1×10^1, H: 4.1×10^3 (arb. units). (b) Mean NIR-II fluorescence intensities corresponding to the fluorescent images in (a). n = 3 biologically independent cells. (c) Mean MSOT intensities corresponding to the optoacoustic images in (a). n = 3 biologically independent cells. Data are presented as mean values +/- SD. F.I.: fluorescence intensity; OA: optoacoustic.

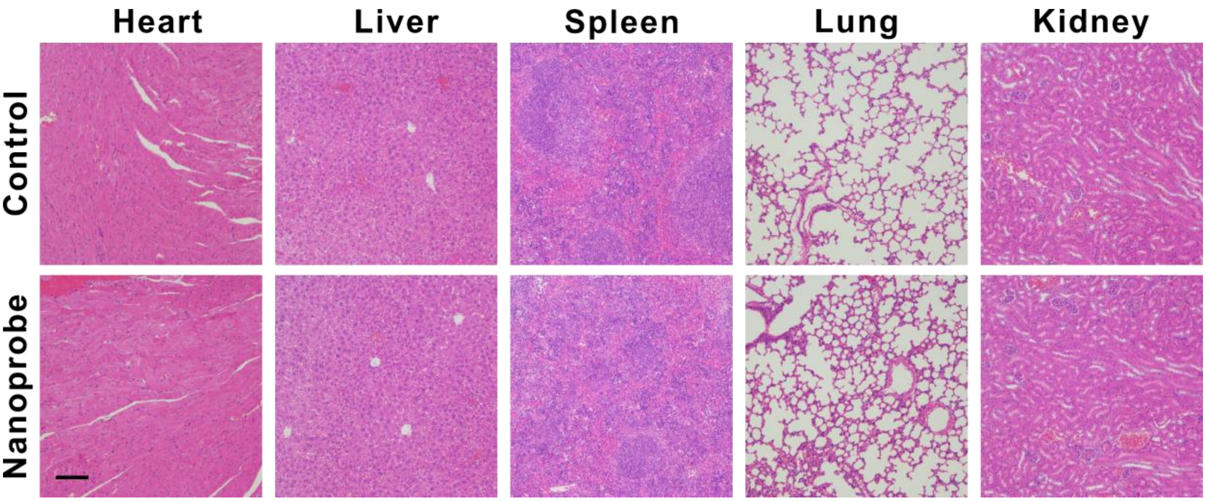

Supplementary Fig. 29. H&E analysis. Representative histological analysis (H&E staining) of heart, liver, spleen, lung and kidney for the healthy mice upon being intravenously injected with saline (the control) or the nanoprobe BTPE-NO_2@F127 (9.1 mg kg^{-1}) for 24 h. n =.5 animals per group. The experiments were repeated independently three times with similar results. Scale bar: 100 μm.

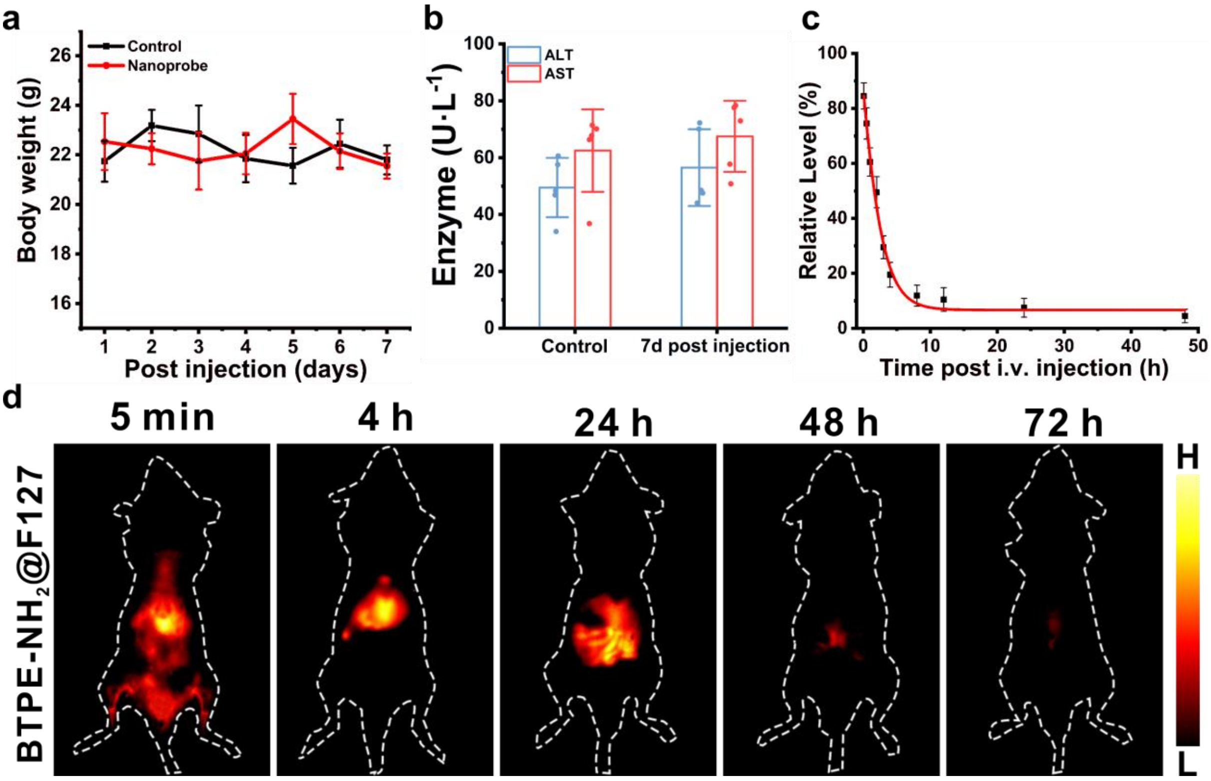

Supplementary Fig. 30. Evaluation of body weights, enzyme levels, pharmacokinetics and biodistribution. (a) Body weights for healthy mice i.v. injected with saline (the control) or the nanoprobe BTPE-NO_2@F127 (9.1 mg kg^{-1}) for 7 days. n = 5 animals per group. Data are presented as mean values +/- SD. (b) Serum levels of two enzymes ALT and AST from the control (healthy mice i.v. injected with saline) and the group 7 days post i.v. injection of nanoprobe. n = 5 animals per group. Data are presented as mean values +/- SD. (c) Blood

retention of BTPE-NH_2@F127 in healthy mice over a span of 48 h post i.v. injection. The injected dose was set to be 100%. First data were collected at 5 min after i.v. injection. Blood was withdrawn at different time points within 48 h post i.v. injection. n = 5 animals per group. Data are presented as mean values +/- SD. (d) NIR-II fluorescent images of healthy mice after i.v. injected with BTPE-NH_2@F127. Color bar: L: 6.0×10^2, H: 6.0×10^4 (arb. units).

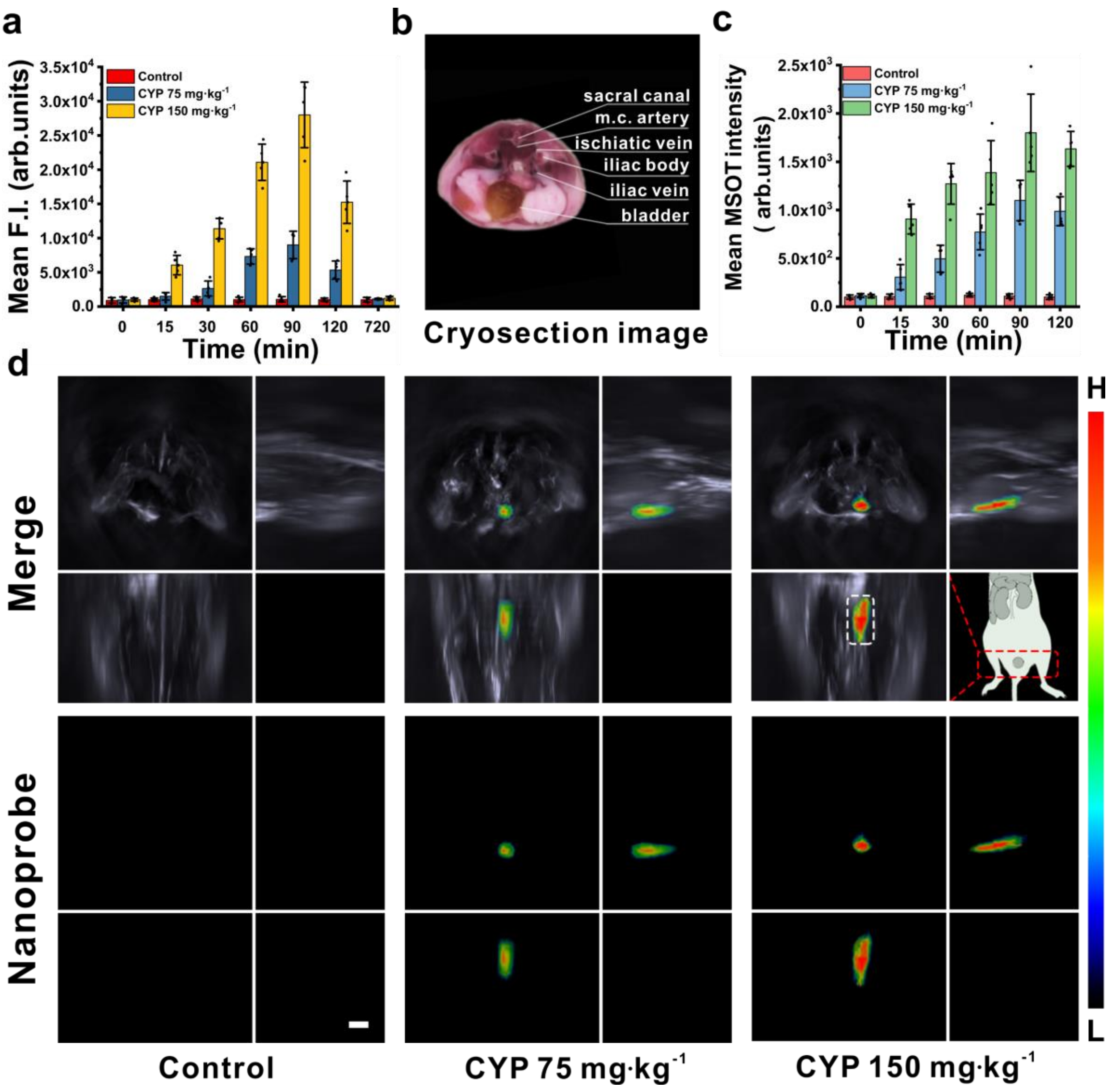

Supplementary Fig. 31. Quantification of NIR-II fluorescent intensity and MSOT intensity, cryosection image and 3D MSOT imaging. (a) Mean NIR-II fluorescent intensities for ROI (blue circle) at bladder in the images in Fig. 3b. n=5 animals per group. Data with error bars are presented as mean ± SD. (b) Representative cryosection image for a female mouse with the location of the cross section corresponding to those in Fig. 4a. The cryosection image is from CryoMOUSETM atlas provided with the MSOT equipment for anatomical reference. (c) Mean MSOT intensities of ROI (white dotted circle) in bladder site in mages of Fig. 4a. n=5 animals per group. Data with error bars are presented as mean ± SD. (d) Representative orthogonal-view 3D MSOT images for the mice pretreated with saline (the control, healthy mice), 75 mg kg^{-1} CYP or 150 mg kg^{-1} CYP at 90 min after intravesical injection of the nanoprobe ($BTPE-NO_2@F127$). Red-dotted rectangle indicates the scanning region. Color bar: L: 6.1×10^1, H: 4.1×10^3 (arb. units). Scale bar: 3 mm. F.I.: fluorescence intensity; MSOT: multispectral optoacoustic tomography.

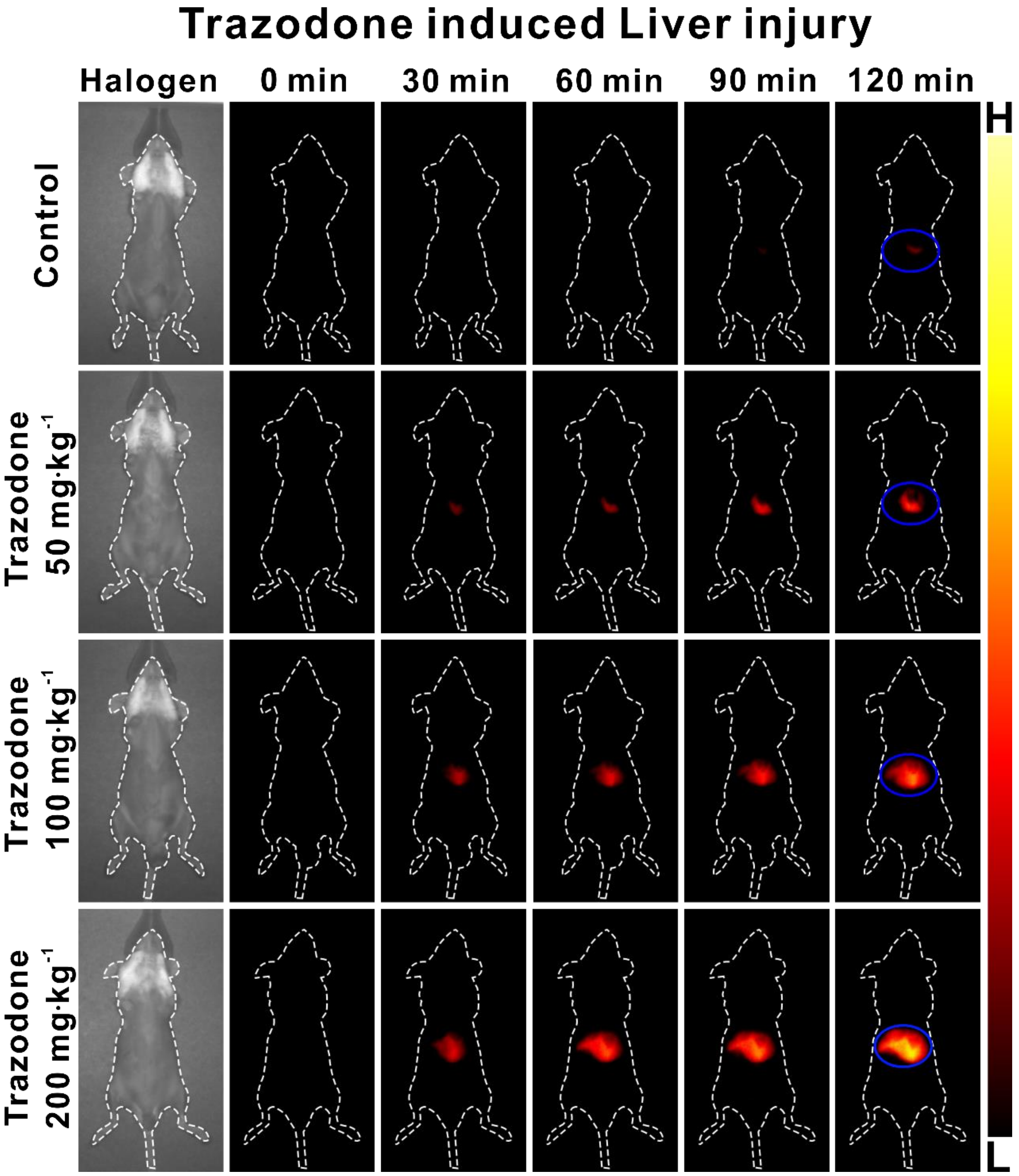

Supplementary Fig. 32. NIR-II fluorescence images of trazodone induced liver injury. Representative NIR-II fluorescence images of the healthy mice pretreated with saline (the control), and the mice pretreated with 50 mg kg^{-1} trazodone, 100 mg kg^{-1} trazodone or 200 mg kg^{-1} trazodone at varied time points upon i.v. injection of the nanoprobe BTPE-NO_2@F127. Blue circle: liver region. Color bar: L: 6.0×10^2, H: 6.0×10^4 (arb. units).

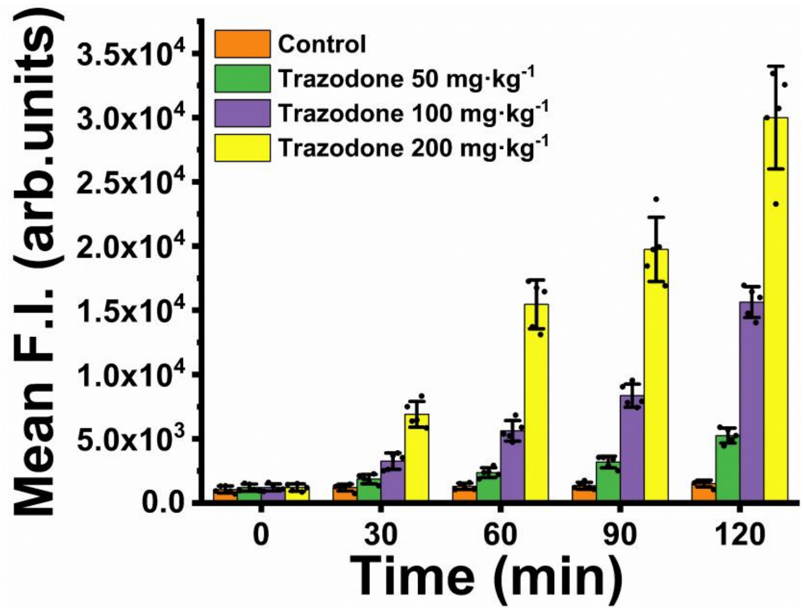

Supplementary Fig. 33. Quantification of NIR-II fluorescence intensity. Mean NIR-II fluorescence intensities at ROI in liver area corresponding to Supplementary Fig. S32. n = 5 animals per group. Data are presented as mean values +/- SD. F.I.: fluorescence intensity.

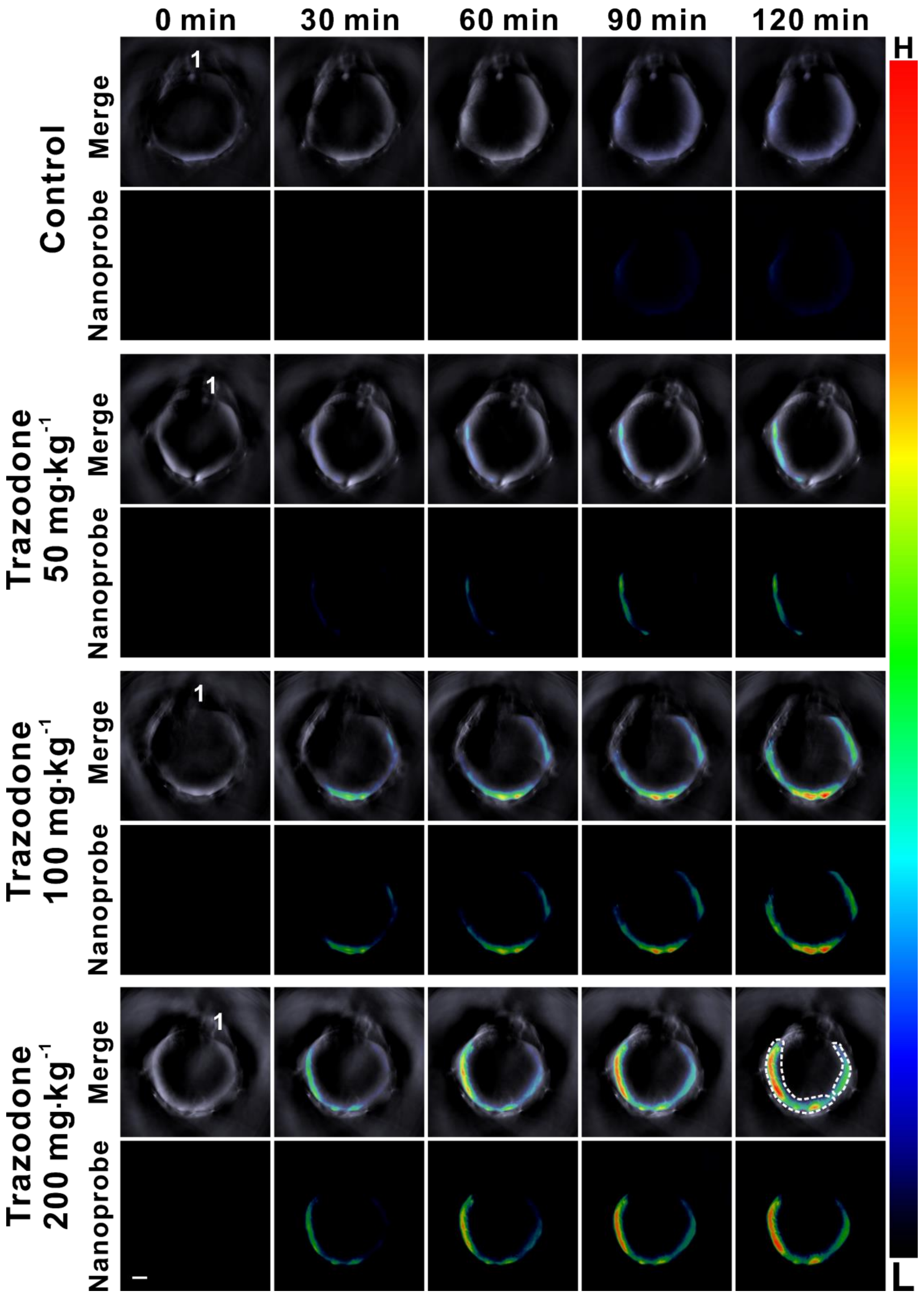

Supplementary Fig. 34. 2D MSOT images of trazodone induced liver injury. Representative cross-sectional MSOT images of mice pretreated with saline (the control), and the mice pretreated with 50 mg kg^{-1} trazodone, 100 mg kg^{-1} trazodone or 200 mg kg^{-1} trazodone at varied time points upon i.v. injection of nanoprobe. Upper panel: Overlay of the activated nanoprobe's signal with the grayscale single-wavelength (850 nm) background image. Organ labeling: 1 Spinal cord. White dotted circle: liver region. Color bar: L: 6.1×10^1, H: 4.1×10^3 (arb. units). Scale bar: 3 mm.

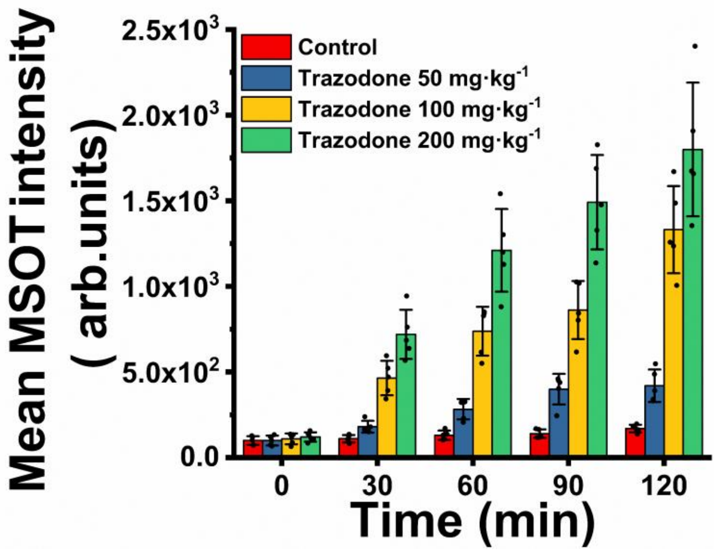

Supplementary Fig. 35. Quantification of MSOT intensity. Mean MSOT intensities for the liver region (ROI) in different mice groups for varied time upon i.v. injection of the nanoprobe BTPE-NO_2@F127 (corresponding to Supplementary Fig. S34). n = 5 animals per group. Data are presented as mean values +/- SD. MSOT: multispectral optoacoustic tomography.

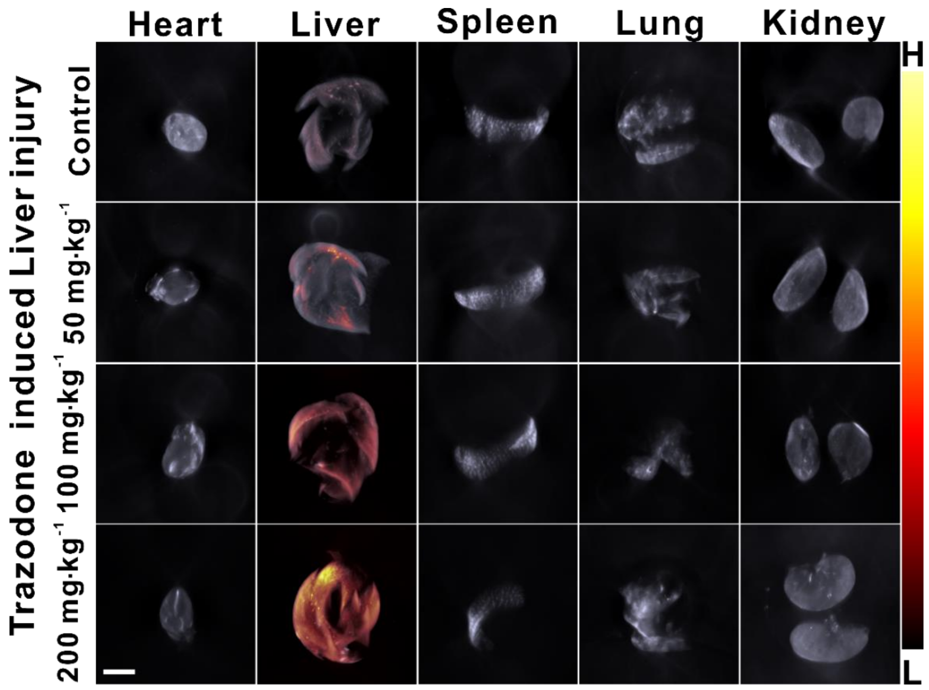

Supplementary Fig. 36. 2D MSOT images of ex vivo organs. Representative cross-sectional MSOT images for the excised main organ from different groups of mice upon pretreatment with saline, 50 mg kg^{-1} trazodone, 100 mg kg^{-1} trazodone or 200 mg kg^{-1} trazodone and then i.v. injection of the nanoprobe BTPE-NO_2@F127 for 120 min. scale bar: 5 mm. Color bar: L: 1.5×10^2, H: 4.1×10^3 (arb. units).

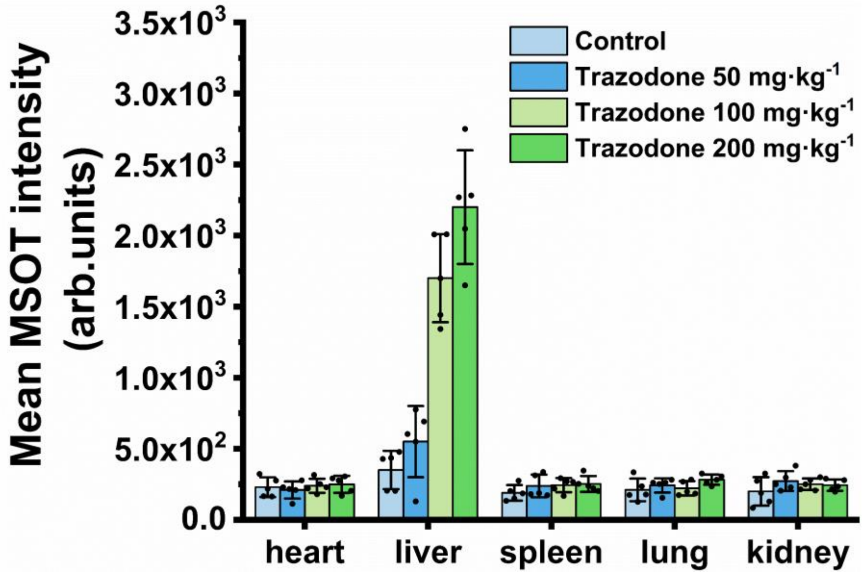

Supplementary Fig. 37. Quantification of MSOT intensity for ex vivo organs. Mean MSOT intensities in major organs corresponding to Supplementary Fig. S36. n = 5 animals per group. Data are presented as mean values +/- SD. MSOT: multispectral optoacoustic tomography.

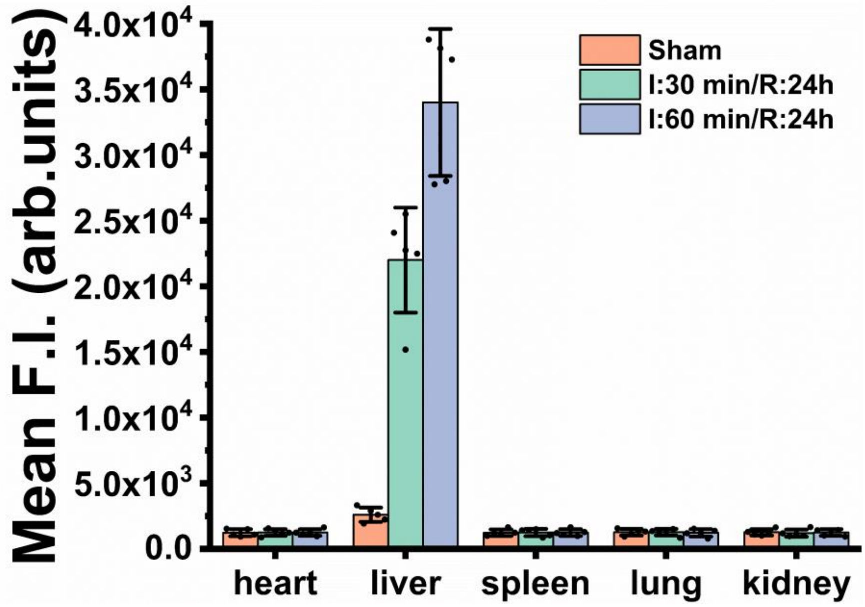

Supplementary Fig. 38. Quantification of NIR-II fluorescence intensity for ex vivo organs. Mean NIR-II fluorescence intensities of major organs corresponding to Fig. 6d. I: ischemia, R: reperfusion. n = 5 animals per group. Data are presented as mean values +/- SD. F.I.: fluorescence intensity. I: ischemia, R: reperfusion.

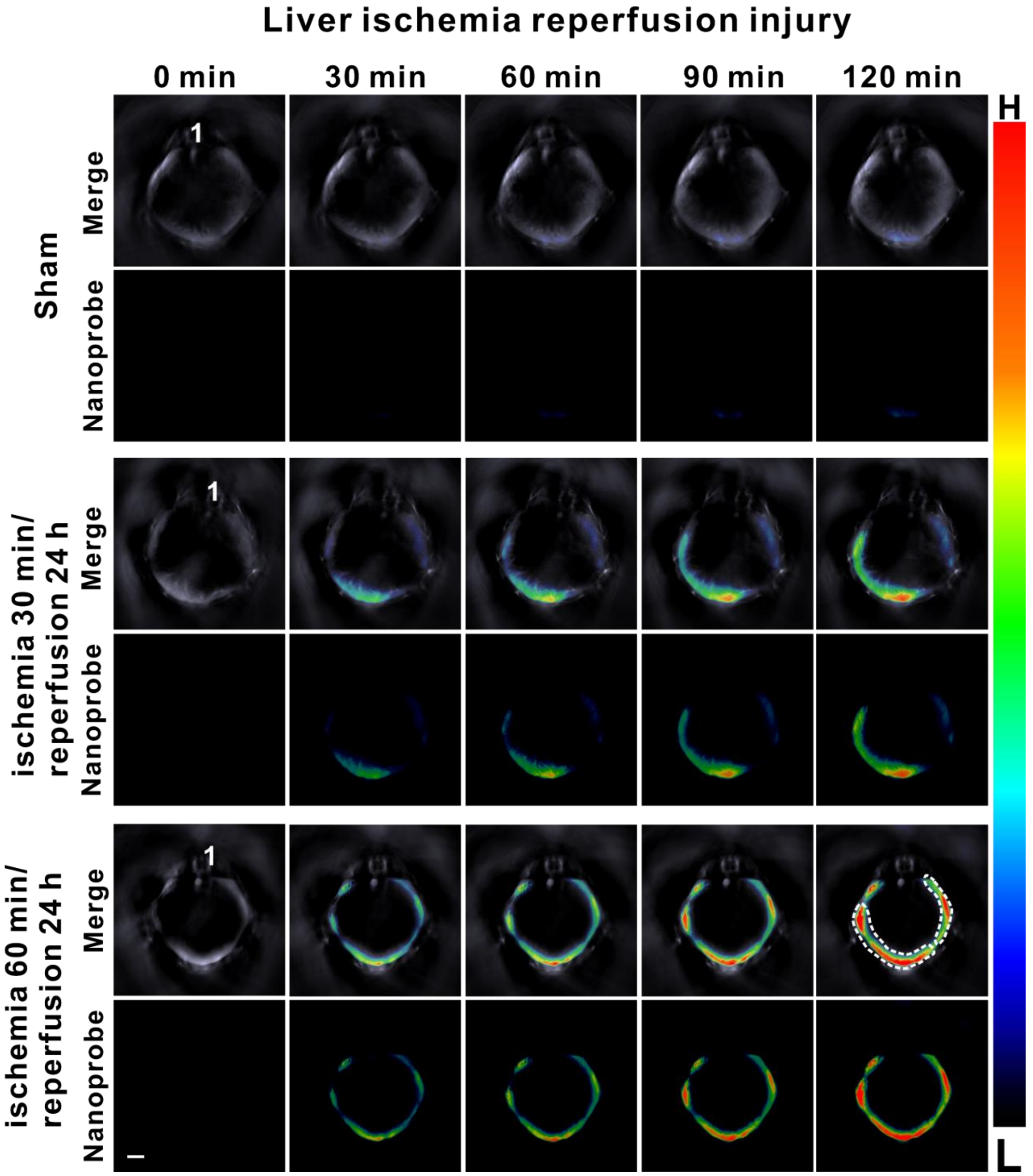

Supplementary Fig. 39. 2D MSOT images of liver ischemia reperfusion injury. Representative cross-sectional MSOT images of the mice pretreated with ischemia for 0 min, 30 min or 60 min and after reperfusion for 24 h at varied time points upon i.v. injection of the nanoprobe BTPE-NO_2@F127. Upper panel: Overlay of the activated probe's signal with the grayscale single-wavelength (850 nm) background image. Organ labeling: 1 Spinal cord. White dotted circle: liver region. Scale bar:3 mm. Color bar: L: 6.1×10^1, H: 4.1×10^3 (arb. units).

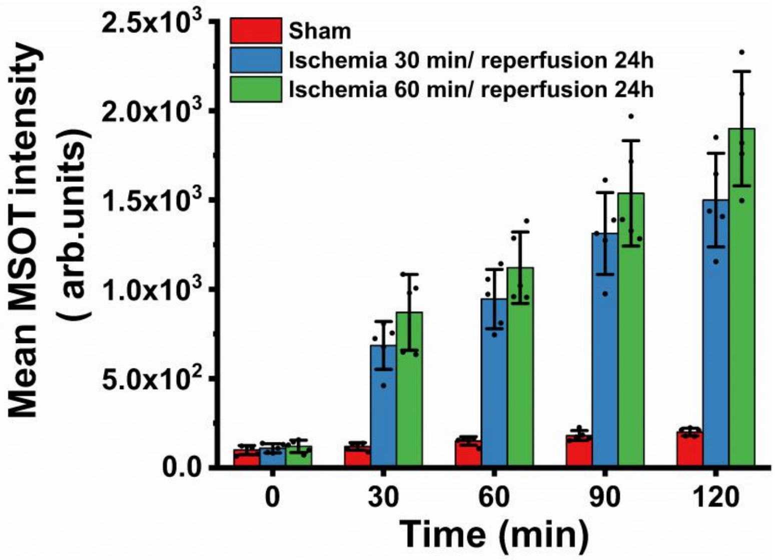

Supplementary Fig. 40. Quantification of MSOT intensity in vivo. Mean MSOT intensities at ROI in the liver region corresponding to Supplementary Fig. S39. n = 5 animals per group. Data are presented as mean values +/- SD. MSOT: multispectral optoacoustic tomography.

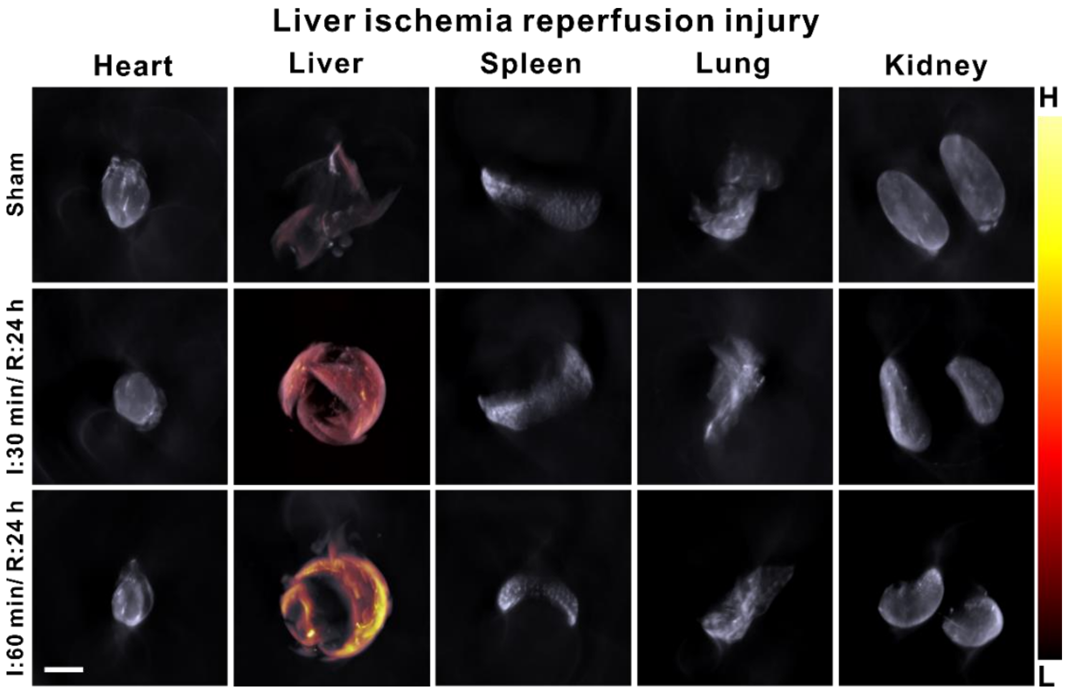

Supplementary Fig. 41. 2D MSOT images of ex vivo organs. Representative cross-sectional MSOT images for excised main organs of the control (sham-surgery, ischemia for 0 min) and the I/R model mice (ischemia for 30 min or 60 min followed by reperfusion for 24 h) at 120 min after i.v. injection of the nanoprobe BTPE-NO_2@F127. Scale bar: 5 mm. Color bar: L: 1.5×10^2, H: 4.1×10^3 (arb. units). I: ischemia, R: reperfusion.

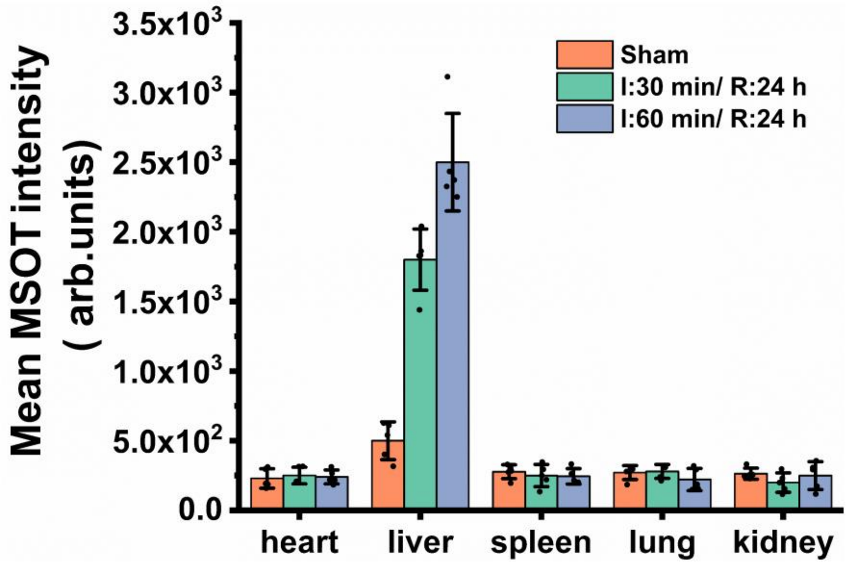

Supplementary Fig. 42. Quantification of MSOT intensity for ex vivo organs. Mean MSOT intensities at major organs corresponding to Supplementary Fig. S41. I: ischemia, R: reperfusion. n = 5 animals per group. Data are presented as mean values +/- SD. MSOT: multispectral optoacoustic tomography.

Supplementary Table 1. Comparation with other organic NIR-II fluorescent systems.

Molecular Structure	AIE/ ACQ	Activatable /Always on	QY (%)	Reference
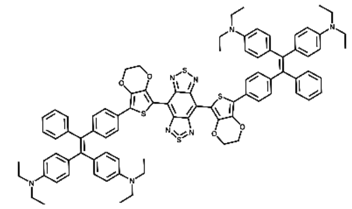 **HLZ-BTED**	AIE	Always on	0.18 HLZ-BTED dots measured in water	1
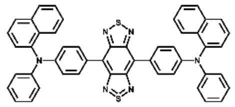 **BPN-BBTD**	AIE	Always on	1.8 BPN-BBTD nanoparticles measured in water	2
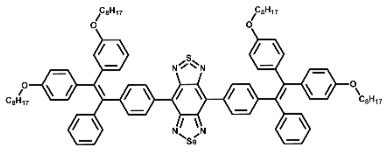 **BPST**	AIE	Always on	5.8 BPST nanoparticles measured in water	3
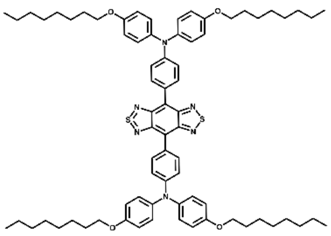 **BPBBT**	AIE	Always on	1.45 BPBBT nanoparticles measured in water	4
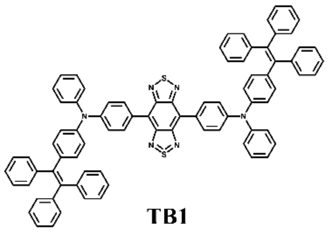 **TB1**	AIE	Always on	6.2 TB1 dots measured in water	5
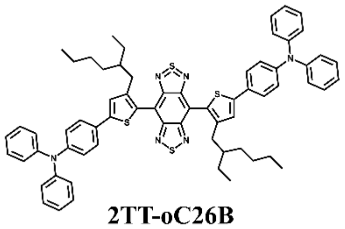 **2TT-oC26B**	AIE	Always on	11.5 2TT-oC26B nanoparticles measured in water	6

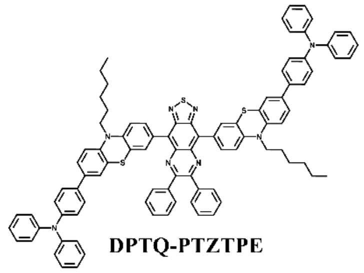 **DPTQ-PTZTPE**	AIE	Always on	0.29<br>DPTQ-PTZTPE nanoparticles measured in water	7
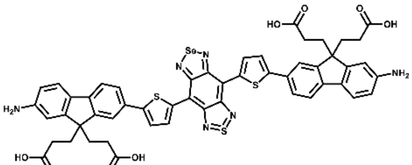 **FM1210**	ACQ	Always on	0.036<br>measured in dichloromethane	8
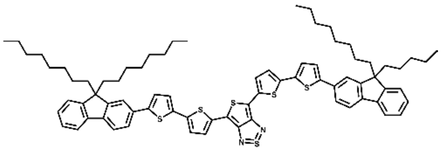 **TTDT-TF**	ACQ	Always on	0.712<br>measured in tetrahydrofuran	9
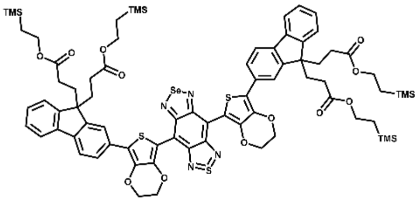 **SY1080**	ACQ	Always on	1.5<br>measured in tetrahydrofuran	10
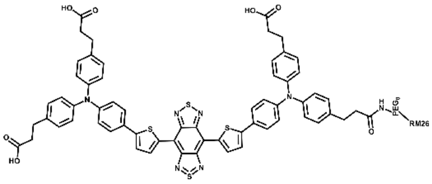 **SCH1100**	ACQ	Always on	0.2<br>measured in water	11
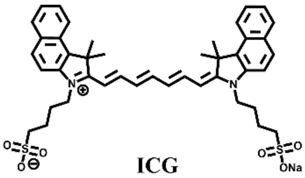 **ICG**<br>(Approved by FDA for human use)	ACQ	Always on	0.9<br>measured in water	12

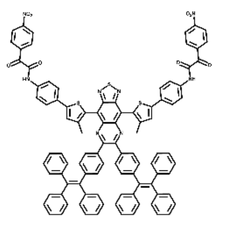<br>**BTPE-NO$_2$**<br>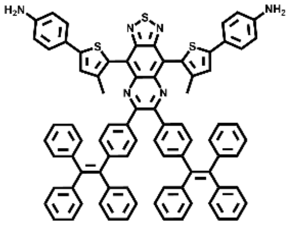<br>**BTPE-NH$_2$**	AIE	Activatable	1.22<br>BTPE-NH$_2$@F127<br>measured in pH 7.4<br>PBS<br>0.73<br>BTPE-NH$_2$<br>measured in pH 7.4<br>PBS containing 5%<br>DMSO	**This work**

QY: fluorescence quantum yield was measured with IR-26 (QY 0.5% in 1,2-dichloroethane) as the reference standard. AIE: aggregation-induced emission. ACQ: aggregation-caused quenching.

Supplementary Table 2. Comparison of probes for NIR fluorescent imaging and optoacoustic (photoacoustic) imaging in the liver injury-related disease models in vivo.

Disease Model	NIR-I or NIR-II FL	SBR for FL	OA	SBR for OA	Activatable / Always on	Reference
Liver ischemia-reperfusion injury	NIR-I	5	/	/	Activatable	13
Liver ischemia-reperfusion injury	NIR-I	3.33	/	/	Activatable	14
INH-induced hepatotoxicity	NIR-I	2.5	OA	4.5	Activatable	15
APAP-induced liver injury	NIR-II	10	/	/	Activatable	16
APAP-induced hepatotoxicity	NIR-II	6	/	/	Activatable	17
Metformin-induced liver injury	NIR-II	5	/	/	Activatable	18
APAP-induced hepatotoxicity	NIR-II	4.5	/	/	Activatable	19
APAP-induced hepatotoxicity	NIR-II	4.4	OA	3.8	Activatable	20
APAP-induced hepatotoxicity	NIR-II	3.3	/	/	Activatable	21
APAP-induced hepatotoxicity	NIR-I	9	OA	4	Activatable	22
CCl_4-induced liver injury	NIR-I	4	OA	1.9	Always on	23
Metformin-induced liver injury	NIR-I	7	OA	9.6	Activatable	24
APAP-induced hepatotoxicity	NIR-I	6	OA	18	Activatable	25
Herbal medicine induced liver injury	NIR-II	6	OA	25	Activatable	26

Trazodone-induced liver injury	NIR-II	20	OA	11	Activatable	**This work**
Liver ischemia-reperfusion injury	NIR-II	15.5	OA	9.5	Activatable	**This work**

The trazodone-induced liver injury has been discovered clinically (e.g., *Am. J. Gastroenterol.* **95**, 532-535 (2000); *Am. J. Psychiatry* **171**, 404-415 (2014); *Ann. Pharmacother.* **35**, 1559-1561 (2001)), but has rarely been studied in animal models. Hence, other drug-induced liver injury models were included for the comparison.

OA: optoacoustic imaging, also known as PAI (photoacoustic imaging). SBR: signal-to-background ratio (determined according to *Angew. Chem. Int. Ed.* **54**, 14758-14762 (2015); *Nat. Commun.* **11**, 3102 (2020)). /: not applicable. APAP: acetaminophen. INH: isoniazid.

Supplementary Table 3. Comparison of probes for NIR fluorescent imaging and optoacoustic (photoacoustic) imaging in bladder-related disease models in vivo.

Disease Model	NIR-I or NIR-II FL	SBR for FL	OA	SBR for OA	Activatable / Always on	Reference
Bladder cancer	NIR-I	10	/	/	Always on	27
Bladder cancer	NIR-I	2.2	/	/	Activatable	28
Bladder cancer	/	/	OA	4.5	Always on	29
Bladder cancer	/	/	OA	14	Always on	30
Bladder cancer	/	/	OA	4.3	Activatable	31
Interstitial Cystitis	NIR-II	28	OA	18	Activatable	**This work**

The NIR-II fluorescence and MSOT imaging of interstitial cystitis or bladder inflammation have rarely been studied in the previous reports. Therefore, the bladder cancer disease models were included for the comparison.

OA: optoacoustic imaging, also known as PAI (photoacoustic imaging); SBR: signal-to-background ratio. /: not applicable.

Supplementary references:

1. Lin, J, et al. Novel near-infrared II aggregation-induced emission dots for in vivo bioimaging. *Chem. Sci.* **10**, 1219-1226 (2019).
2. Alifu, N. et al. Single-molecular near-infrared-II theranostic systems: ultrastable aggregation-induced emission nanoparticles for long-term tracing and efficient photothermal therapy. *ACS Nano* **12**, 11282-11293 (2018).
3. Wu, W. et al. Molecular engineering of an organic NIR-II fluorophore with aggregation-induced emission characteristics for in vivo imaging. *Small* **15**, 1805549 (2019).
4. Gao, S. et al. Albumin tailoring fluorescence and photothermal conversion effect of near-infrared-II fluorophore with aggregation-induced emission characteristics. *Nat. Commun.* **10**, 2206 (2019).
5. Sheng, Z. et al. Bright aggregation-induced-emission dots for targeted synergetic NIR-II fluorescence and NIR-I photoacoustic imaging of orthotopic brain tumors. *Adv. Mater.* **30**, 1800766 (2018).
6. Li, Y. et al. Design of AIEgens for near-infrared IIb imaging through structural modulation at molecular and morphological levels. *Nat. Commun.* **11**, 1255 (2020).
7. Li, S. et al. Second near-infrared aggregation-induced emission fluorophores with phenothiazine derivatives as the donor and 6,7-diphenyl-[1,2,5] thiadiazolo [3,4-g] quinoxaline as the acceptor for in vivo imaging. *ACS Appl. Mater. Interfaces* **12**, 20281-20286 (2020).
8. Fang, Y. et al. Design, synthesis, and application of a small molecular NIR-II fluorophore with maximal emission beyond 1200 nm. *J. Am. Chem. Soc.* **142**, 15271-15275 (2020).
9. Sun, P. et al. Thienothiadiazole-based NIR-II dyes with D–A–D structure for NIR-II fluorescence imaging systems. *ACS Appl. Bio Mater.* **4**, 4542-4548 (2021).
10. Zhang, R. et al. Rational design of a multifunctional molecular dye with single dose and laser for efficiency NIR-II fluorescence/photoacoustic imaging guided photothermal therapy. *Anal. Chem.* **91**, 12476-12483 (2019).
11. Sun, Y. et al. Novel benzo-bis(1,2,5-thiadiazole) fluorophores for in vivo NIR-II imaging of cancer. *Chem. Sci.* **7**, 6203-6207 (2016).
12. Carr, J. A. et al. Shortwave infrared fluorescence imaging with the clinically approved near-infrared dye indocyanine green. *Proc. Natl. Acad. Sci. U.S.A.* **115**, 4465-4470 (2018).
13. Zhang, W. et al. Two-photon fluorescence imaging of mitochondrial superoxide anion transport mediating liver ischemia-reperfusion injury in mice. *Chem. Commun.* **55**, 10740-10743 (2019).
14. Zhang, W. et al. In situ and real-time imaging of superoxide anion and peroxynitrite elucidating arginase 1 nitration aggravating hepatic ischemia-reperfusion injury. *Biomaterials* **225**, 119499 (2019).
15. Fan, X. et al. Activatable photoacoustic/fluorescent dual-modal probe for monitoring of

drug-induced liver hypoxia in vivo. *Chem. Commun.* **57**, 8644-8647 (2021).
16. Tang, Y. et al. Organic semiconducting nanoprobe with redox-activatable NIR-II fluorescence for in vivo real-time monitoring of drug toxicity. *Chem. Commun.* **55**, 27-30 (2019).
17. Li, D. et al. Peroxynitrite activatable NIR-II fluorescent molecular probe for drug-induced hepatotoxicity monitoring. *Anal. Chem.* **91**, 4771-4779 (2019).
18. Deng, Z. et al. Endogenous H_2S-activated orthogonal second near-infrared emissive nanoprobe for in situ ratiometric fluorescence imaging of metformin-induced liver injury. *ACS Nano* **15**, 3201-3211 (2021).
19. Lei, Z. et al. Stable, wavelength-tunable fluorescent dyes in the NIR-II region for in vivo high-contrast bioimaging and multiplexed biosensing. *Angew. Chem. Int. Ed.* **58**, 8166-8171 (2019).
20. Zhang, X. et al. Plasmonic-fluorescent Janus Ag/Ag_2S nanoparticles for in situ H_2O_2-activated NIR-II fluorescence imaging. *Nano Lett.* **21**, 2625-2633 (2021).
21. Ren, T. et al. A general strategy for development of activatable NIR-II fluorescent probes for in vivo high-contrast bioimaging. *Angew. Chem. Int. Ed.* **60**, 800-805 (2021).
22. Zhuang, H. et al. Real-time monitoring and accurate diagnosis of drug-induced hepatotoxicity in vivo by ratio-fluorescence and photoacoustic imaging of peroxynitrite. *Nanoscale* **12**, 10216-10225 (2020).
23. Zhou, Z. et al. ZnDPA-conjugated cyanine probes for targeted near-infrared fluorescence and photoacoustic imaging of drug-induced liver injury in vivo. *Dyes Pigm.* **194**, 109586 (2021).
24. Sun, L. et al. A turn-on optoacoustic probe for imaging metformin-induced upregulation of hepatic hydrogen sulfide and subsequent liver injury. *Theranostics* **9**, 77 (2019).
25. Wu, Y. et al. Activatable probes for diagnosing and positioning liver injury and metastatic tumors by multispectral optoacoustic tomography. *Nat. Commun.* **9**, 3983 (2018).
26. Sun, L. et al. An activatable probe with aggregation-induced emission for detecting and imaging herbal medicine induced liver injury with optoacoustic imaging and NIR-II fluorescence imaging. *Adv. Healthcare Mater*. (2021) DOI: 10.1002/adhm.202100867.
27. Li, G. et al. Fluorinated polyethylenimine to enable transmucosal delivery of photosensitizer-conjugated catalase for photodynamic therapy of orthotopic bladder tumors postintravesical instillation. *Adv. Funct. Mater.* **29**, 1901932 (2019).
28. Huang, J. et al. A renal-clearable macromolecular reporter for near-infrared fluorescence imaging of bladder cancer. *Angew. Chem. Int. Ed.* **59**, 4415-4420 (2020).
29. Nguyen, V. P. et al. Feasibility of photoacoustic evaluations on dual-thermal treatment of ex vivo bladder tumors. *J. Biophotonics* **10**, 577-588 (2017).
30. Zhang, D. et al. High-performance identification of human bladder cancer using a signal self-amplifiable photoacoustic nanoprobe. *ACS Appl. Mater. Interfaces* **10**, 28331-28339 (2018).

31. Wu, Y. et al. A nanoprobe for diagnosing and mapping lymphatic metastasis of tumor using 3D multispectral optoacoustic tomography owing to aggregation/deaggregation induced spectral change. *Adv. Funct. Mater.* **29**, 1807960 (2019).
