# Supplementary material for: A H2O2-activatable nanoprobe for diagnosing interstitial cystitis and liver ischemia-reperfusion injury via multispectral optoacoustic tomography and NIR-II fluorescent imaging
Source: Nat Commun. 2021 Nov 25;12:6870. doi: 10.1038/s41467-021-27233-4 (PMC8617030; doi:10.1038/s41467-021-27233-4)
Supplement: Supplementary file 3 — Reporting Summary [file 41467_2021_27233_MOESM3_ESM.pdf]

nature portfolio

Corresponding author(s): Yanli Zhao; Shuizhu Wu; Fang Zeng.

Last updated by author(s): Oct 30, 2021

Reporting Summary

Nature Portfolio wishes to improve the reproducibility of the work that we publish. This form provides structure for consistency and transparency in reporting. For further information on Nature Portfolio policies, see our Editorial Policies and the Editorial Policy Checklist.

Statistics

For all statistical analyses, confirm that the following items are present in the figure legend, table legend, main text, or Methods section.

n/a	Confirmed	
☐	☒	The exact sample size (*n*) for each experimental group/condition, given as a discrete number and unit of measurement
☐	☒	A statement on whether measurements were taken from distinct samples or whether the same sample was measured repeatedly
☒	☐	The statistical test(s) used AND whether they are one- or two-sided<br>*Only common tests should be described solely by name; describe more complex techniques in the Methods section.*
☒	☐	A description of all covariates tested
☒	☐	A description of any assumptions or corrections, such as tests of normality and adjustment for multiple comparisons
☐	☒	A full description of the statistical parameters including central tendency (e.g. means) or other basic estimates (e.g. regression coefficient) AND variation (e.g. standard deviation) or associated estimates of uncertainty (e.g. confidence intervals)
☒	☐	For null hypothesis testing, the test statistic (e.g. *F*, *t*, *r*) with confidence intervals, effect sizes, degrees of freedom and *P* value noted<br>*Give P values as exact values whenever suitable.*
☒	☐	For Bayesian analysis, information on the choice of priors and Markov chain Monte Carlo settings
☒	☐	For hierarchical and complex designs, identification of the appropriate level for tests and full reporting of outcomes
☒	☐	Estimates of effect sizes (e.g. Cohen's *d*, Pearson's *r*), indicating how they were calculated

Our web collection on statistics for biologists contains articles on many of the points above.

Software and code

Policy information about availability of computer code

Data collection	Data of fluorescent spectra were collected with software of SpectraSuite 1.6.0_11. Data of animal imaging were collected with PSL Viewer v4.7.5 of NIR-II in Vivo Imaging System (Series II 808/900-1700, Suzhou NIR-Optics Technologies Co., Ltd.) and viewMSOT 3.8 of inVision128 multispectral optoacoustic tomographic (MSOT) imaging system (iThera Medical GmbH).
Data analysis	NMR spectra were analyzed using Mestre Nova LITE v14.0.0.0-23239 software (Mestre lab Research S.L.). Statistical calculations were performed using OriginPro 2018 (64 bit) SR1 b9.5.1.195. Data analysis of absorption spectra and fluorescence spectra was performed using OriginPro 2018 (64 bit) SR1 b9.5.1.195. Calculations were carried out using Gaussian 16 W. Data analysis of animal imaging was conducted using viewMSOT 3.8 and PSLViewer v4.7.5.

For manuscripts utilizing custom algorithms or software that are central to the research but not yet described in published literature, software must be made available to editors and reviewers. We strongly encourage code deposition in a community repository (e.g. GitHub). See the Nature Portfolio guidelines for submitting code & software for further information.

Data

Policy information about availability of data

All manuscripts must include a data availability statement. This statement should provide the following information, where applicable:

- Accession codes, unique identifiers, or web links for publicly available datasets
- A description of any restrictions on data availability
- For clinical datasets or third party data, please ensure that the statement adheres to our policy

The authors declare that the data supporting the findings of this study are available within the article and its Supplementary Information. Extra data are available from the corresponding authors upon reasonable request.

Field-specific reporting

Please select the one below that is the best fit for your research. If you are not sure, read the appropriate sections before making your selection.

☒ Life sciences ☐ Behavioural & social sciences ☐ Ecological, evolutionary & environmental sciences

For a reference copy of the document with all sections, see nature.com/documents/nr-reporting-summary-flat.pdf

Life sciences study design

All studies must disclose on these points even when the disclosure is negative.

Sample size	Sample size was chosen based on previous experience with the animal models or following convention of the methods. Sample size was determined as n≥ 3. As the nature of the experiments was exploratory, the number of individuals in each experiment was determined to validate technological advance and was not used to validate a biological hypothesis.
Data exclusions	No data was excluded from the data analysis.
Replication	Experiments were repeated at least three independent experiments with similar results. All experiments were reproduced to reliably support conclusions stated in the manuscript.
Randomization	Allocation was random.
Blinding	The investigators were blinded to group allocation during data collection and analysis.

Reporting for specific materials, systems and methods

We require information from authors about some types of materials, experimental systems and methods used in many studies. Here, indicate whether each material, system or method listed is relevant to your study. If you are not sure if a list item applies to your research, read the appropriate section before selecting a response.

Materials & experimental systems

n/a	Involved in the study	
☒	☐	Antibodies
☐	☒	Eukaryotic cell lines
☒	☐	Palaeontology and archaeology
☐	☒	Animals and other organisms
☒	☐	Human research participants
☒	☐	Clinical data
☒	☐	Dual use research of concern

Methods

n/a	Involved in the study	
☒	☐	ChIP-seq
☒	☐	Flow cytometry
☒	☐	MRI-based neuroimaging

Eukaryotic cell lines

Policy information about cell lines

Cell line source(s)	RAW264.7 was obtained from KeyGen Biology Co. Ltd (Nanjing, China)
Authentication	The suppliers routinely authenticate the cell lines by short tandem repeat profiling, and the cell lines were not authenticated by our laboratory.
Mycoplasma contamination	Mycoplasma test was negative.
Commonly misidentified lines (See ICLAC register)	No commonly misidentified cell lines was used

Animals and other organisms

Policy information about studies involving animals; ARRIVE guidelines recommended for reporting animal research

Laboratory animals	The male and female BALB/C mice (6-7 weeks old) were purchased from Guangdong Medical Laboratory Animal Center (GDMLAC) and kindly kept in the Laboratory Animal Center of South China Agricultural University.
Wild animals	The study did not involve wild animals.
Field-collected samples	No field-collected samples was used.

Ethics oversight	The in vivo experiments were approved and conducted in compliance with the regulations of Ethics Committee of Laboratory Animal Center of South China Agricultural University (Approval No.: 2020-d087).

Note that full information on the approval of the study protocol must also be provided in the manuscript.
